# GSG1L suppresses AMPA receptor-mediated synaptic transmission and uniquely modulates AMPA receptor kinetics in hippocampal neurons

**DOI:** 10.1038/ncomms10873

**Published:** 2016-03-02

**Authors:** Xinglong Gu, Xia Mao, Marc P. Lussier, Mary Anne Hutchison, Liang Zhou, F. Kent Hamra, Katherine W. Roche, Wei Lu

**Affiliations:** 1 Synapse and Neural Circuit Research Unit, National Institute of Neurological Disorders and Stroke, National Institutes of Health, 35 Convent Drive, 3C1000, Bethesda, Maryland 20892, USA; 2 Receptor Biology Section, National Institute of Neurological Disorders and Stroke, National Institutes of Health, 35 Convent Drive, 2C903, Bethesda, Maryland 20892, USA; 3 Department of Pharmacology, Cecil H. & Ida Green Center for Reproductive Biology Sciences, University of Texas Southwestern Medical Center in Dallas, Dallas, Texas 75390, USA

## Abstract

Regulation of AMPA receptor (AMPAR)-mediated synaptic transmission is a key mechanism for synaptic plasticity. In the brain, AMPARs assemble with a number of auxiliary subunits, including TARPs, CNIHs and CKAMP44, which are important for AMPAR forward trafficking to synapses. Here we report that the membrane protein GSG1L negatively regulates AMPAR-mediated synaptic transmission. Overexpression of GSG1L strongly suppresses, and GSG1L knockout (KO) enhances, AMPAR-mediated synaptic transmission. GSG1L-dependent regulation of AMPAR synaptic transmission relies on the first extracellular loop domain and its carboxyl-terminus. GSG1L also speeds up AMPAR deactivation and desensitization in hippocampal CA1 neurons, in contrast to the effects of TARPs and CNIHs. Furthermore, GSG1L association with AMPARs inhibits CNIH2-induced slowing of the receptors in heterologous cells. Finally, GSG1L KO rats have deficits in LTP and show behavioural abnormalities in object recognition tests. These data demonstrate that GSG1L represents a new class of auxiliary subunit with distinct functional properties for AMPARs.

## Abstract (web summary)

The molecular mechanism controlling the trafficking and function of AMPARs at synapses are not fully understood. Here the authors show that GSG1L, a membrane protein, negatively regulates AMPAR-mediated synaptic transmission and represents a new class of AMPAR auxiliary subunit.

## Graphical abstract

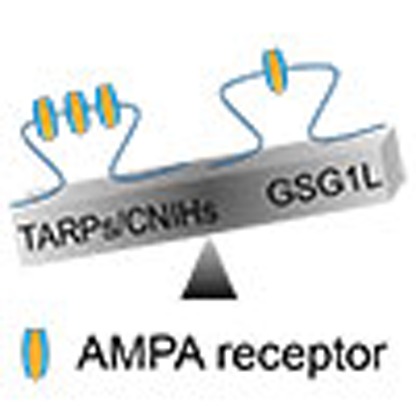

Glutamate is the predominant excitatory neurotransmitter in the central nervous system and acts on AMPA-, Kainate- and NMDA-type ionotropic glutamate receptors to mediate excitatory synaptic transmission^1^. Among them, AMPA receptors (AMPARs) mediate the majority of fast excitatory synaptic transmission in the brain. Accumulating evidence indicates that changes in synaptic strength associated with synaptic plasticity are due in large part to changes in the abundance and kinetic properties of AMPARs at the postsynaptic density^2^,^3^,^4^,^5^. Thus, elucidation of the mechanisms underlying the dynamic modulation of AMPAR trafficking and function at synapses will be the key to understanding the regulation of synaptic strength in the brain. Although substantial progress has been made during the past two decades, not all of the mechanisms for the regulation of the trafficking and function of AMPARs at synapses are fully understood.

A number of transmembrane proteins in the mammalian brain have been reported to bind to AMPARs, including transmembrane AMPAR regulatory proteins (TARPs), Cornichon 2/3 (CNIH2/3), Cystine-knot AMPAR modulating protein (CKAMP44), SynDig1 and Germ Cell-Specific Gene 1-Like (GSG1L)^6^,^7^,^8^,^9^,^10^,^11^,^12^,^13^,^14^,^15^,^16^. Among these, TARPs and CNIH2/3 are the best characterized and clearly regulate AMPAR trafficking and kinetic properties in both heterologous cells and neurons^17^,^18^,^19^. Indeed, gene knockout (KO)/knock-in experiments demonstrate that both γ8, the dominant TARP in the hippocampus, and CNIHs are required for AMPAR forward trafficking to the neuronal surface and synapses in hippocampal CA1 pyramidal neurons^10^,^20^,^21^. In addition, both γ8 and CNIH2/3 modulate AMPAR biophysical properties by slowing receptor deactivation and desensitization kinetics^7^,^10^,^13^,^17^,^22^,^23^,^24^,^25^,^26^,^27^,^28^,^29^. In addition, while TARPs modulate the receptor recovery from desensitization^27^,^30^,^31^,^32^, CNIHs have no effect^7^. More recently, CKAMP44 has also been shown to play an important role in positively regulating AMPAR trafficking to synapses and slowing the receptor deactivation kinetics in hippocampal neurons^15^. These studies indicate that a general function for TARPs/CNIHs/CKAMP44 in the hippocampus is to positively regulate AMPAR abundance at synapses and render slower glutamatergic currents.

Recently, through proteomic screening, GSG1L was found to be present in AMPAR complexes in the brain^8^,^9^. In heterologous cells, GSG1L slows AMPAR deactivation and desensitization similar to TARPs^8^,^9^. In addition, GSG1L is localized at glutamatergic synapses, suggesting a potential role for GSG1L in the regulation of excitatory synaptic transmission^8^,^9^. However, its physiological role in neurons remains unknown. Here we have employed electrophysiology with overexpression and gene inactivation approaches to show that GSG1L negatively regulates AMPAR abundance at synapses and speeds up deactivation and desensitization kinetics of AMPARs in hippocampal CA1 pyramidal neurons. In addition, GSG1L is important for the regulation of long-term potentiation (LTP) and for non-spatial novel object recognition memory. These findings reveal unique roles of GSG1L in the regulation of AMPAR-mediated synaptic transmission in the brain.

## Results

### GSG1L overexpression suppresses AMPA EPSCs in CA1 neurons

**Figure 1:**
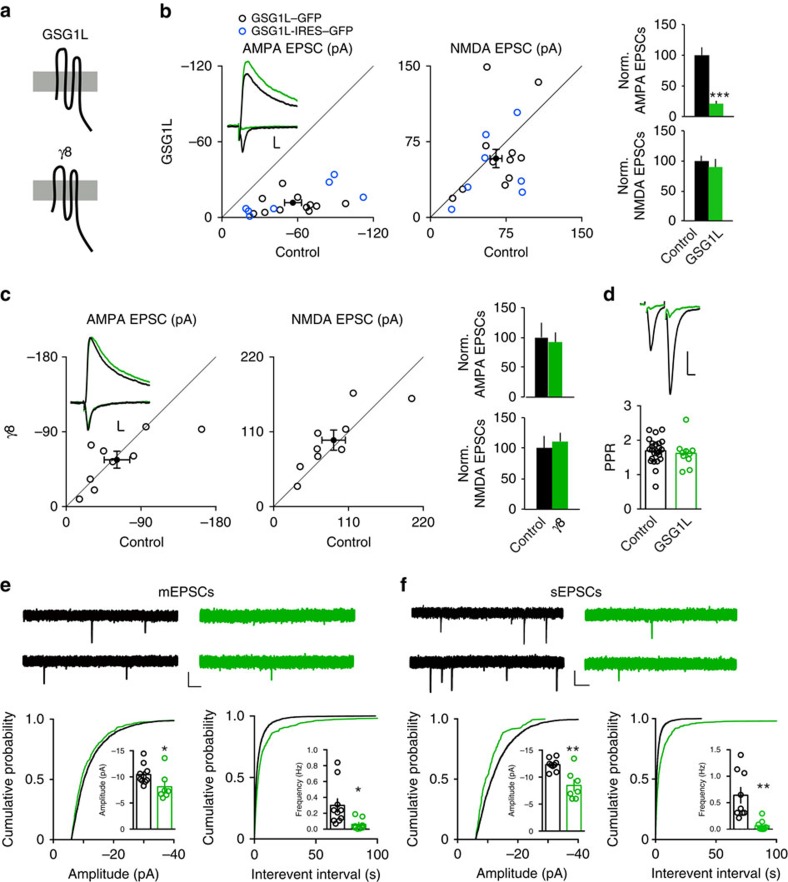
Overexpression of GSG1L in CA1 pyramidal neurons strongly reduced AMPA EPSCs. (**a**) Schematic of γ8 and GSG1L. (**b**,**c**) Overexpression of GSG1L (AMPA, *n*=18; *P*<0.001; NMDA, *n*=18; *P*=0.84; paired *t*-test), but not γ8 (AMPA, *n*=9; *P*=0.66; NMDA, control, *n*=9; *P*=0.35; paired *t*-test) in cultured organotypic hippocampal slices significantly reduced AMPA, but not NMDA EPSCs, in CA1 pyramidal neurons. Note that we used two different constructs to express GSG1L (GSG1L–GFP, black open circles and GSG1L-IRES–GFP, blue open circles in the scatter plots in **b**; There was no significant difference between the two constructs; The solid circle represents average of all pair recordings). Scale bar, 20 pA and 20 ms. (**d**) There was no change of paired-pulse ratio (PPR) in neurons expressing GSG1L (control, *n*=24; GSG1L, *n*=10; *P*=0.61; *t*-test). Scale bar, 50 pA and 20 ms. (**e**) mEPSC recordings showed that there were significant reductions of both mEPSC amplitude and frequency (Amplitude: control, *n*=10; GSG1L: *n*=7; *P*<0.05; Frequency: control, *n*=10; GSG1L, *n*=8; *P*<0.05; *t*-test; Kolmogorov–Smirnov test was used for cumulative distributions, *P*<0.001). Scale bar, 10 pA and 500 ms. (**f**) sEPSC recordings showed that there were significant reductions of both sEPSC amplitude and frequency (Amplitude: control, *n*=9; GSG1L, *n*=7; *P*<0.01; Frequency: control, *n*=9; GSG1L, *n*=9; *P*<0.01; *t*-test; Kolmogorov–Smirnov test was used for cumulative distributions, *P*<0.001). Scale bar, 10 pA and 500 ms. Statistical significance is presented as *P<0.05, **P<0.01 or ***P<0.001.

We first employed a co-immunoprecipitation (co-IP) experiment to evaluate GSG1L binding to the AMPAR GluA1 subunit, but not Kainate receptor GluK1 subunit, in HEK cells and observed robust co-IP between GluA1 and GSG1L as previously reported (Supplementary Figs 1a,b and 14) (refs 8, 9). GSG1L is expressed at excitatory synapses in the hippocampal CA3 region^8^,^9^ and is also expressed in dendritic spines of CA1 pyramidal neurons (B. Fakler, Univ. Freiburg, personal communication). To study the role of GSG1L in the regulation of AMPAR-mediated synaptic transmission, we biolistically transfected cultured organotypic hippocampal slices with gold particles that were coated with plasmids expressing GSG1L fused to green fluorescent protein (GSG1L–GFP) or GSG1L-IRES–GFP. After 2–4 days, we performed simultaneous dual whole-cell voltage clamp recordings that were made from a transfected CA1 pyramidal cell (that is, identified by green fluorescent protein (GFP) signal) and a neighbouring control cell to measure AMPA and NMDA excitatory postsynaptic currents (EPSCs). A single stimulating electrode was used to evoke EPSCs in both cells, so that the effects of the postsynaptic manipulation in the transfected cell could be compared with the normal neighbouring cell. Expression of GSG1L specifically reduced the amplitude of AMPA EPSCs by ∼80%, without affecting NMDA EPSC amplitudes (Fig. 1a,b). Importantly, in agreement with a previous report^20^, overexpression of γ8, which shares a similar topology with GSG1L, in CA1 pyramidal neurons, did not affect either AMPA or NMDA EPSCs (Fig. 1a,c). To explore further the specificity of the effect of GSG1L on AMPA EPSCs, we expressed Slitrk3, a transmembrane protein important for the development of GABAergic synapses, in hippocampal slice cultures and found that there was no significant impact on AMPA and NMDA EPSCs (Supplementary Fig. 2). There was no change of paired-pulse ratio (PPR) (Fig. 1d), a measure of presynaptic neurotransmitter release probability in neurons overexpressing GSG1L. Furthermore, miniature EPSC (mEPSC) or spontaneous EPSC (sEPSC) analysis in GSG1L-overexpressing neurons in the presence or absence of tetrodotoxin (TTX), respectively, showed that there was a strong reduction of m/sEPSC frequency and a significant decrease of m/sEPSC amplitude (Fig. 1e,f). Analysis of spine density in cells expressing GSG1L demonstrated that there was no difference between GSG1L-expressing cells and control cells in the CA1 region in organotypic slice cultures (Supplementary Fig. 3). Together with the data that showed the lack of changes of NMDA EPSCs, PPR and spine density in neurons overexpressing GSG1L (Fig. 1b,d and Supplementary Fig. 3), AMPA EPSC and m/sEPSC data indicate that overexpression of GSG1L induced a loss of functional AMPARs at the majority of synapses (that is, strong decrease of m/sEPSC frequency) and a reduced amount of functional AMPARs at the remaining synapses (that is, significant decrease of m/sEPSC amplitude) (Fig. 1b,e,f). Collectively, these data show that overexpression of GSG1L strongly and specifically impairs AMPAR-mediated synaptic transmission.

**Figure 2:**
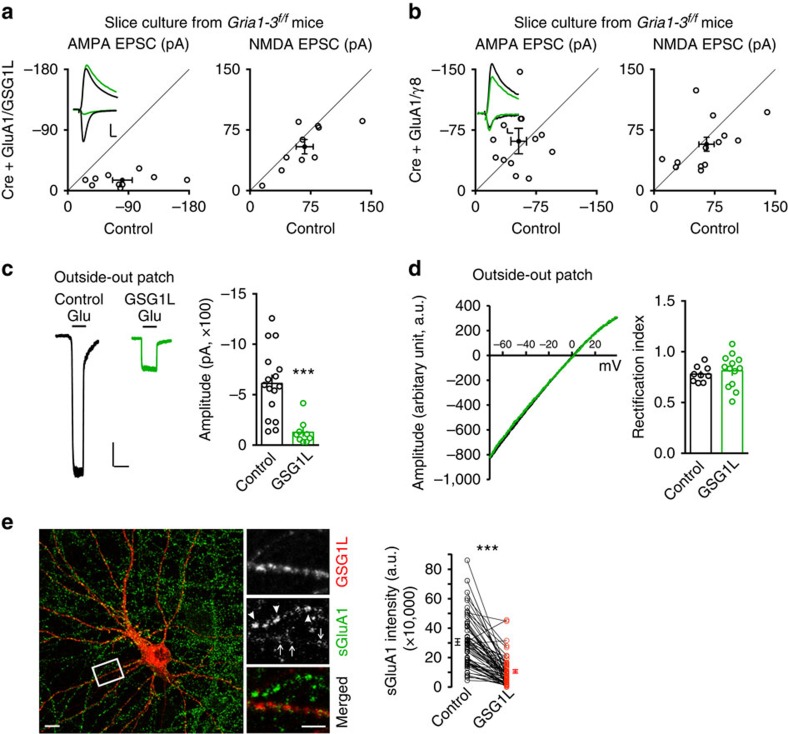
Overexpression of GSG1L impaired AMPAR trafficking to synaptic and extrasynaptic membranes. (**a**,**b**) While GluA1/γ8 fully rescued AMPA EPSCs (**b**, AMPA, *n*=13; *P*=0.51; paired *t*-test; Scale bar, 20 pA and 20 ms), GluA1/GSG1L (**a**, AMPA, *n*=10; *P*<0.01; paired *t*-test; Scale bar, 50 pA and 20 ms) only rescued ∼20% of AMPA EPSCs in CA1 pyramidal neurons expressing Cre in cultured organotypic hippocampal slices prepared from *Gria1–3*^*f/f*^ mice. (**c**) Overexpression of GSG1L strongly reduced AMPAR-mediated somatic outside-out patch currents induced by 1 mM glutamate in the presence of 100 μM cyclothiazide (control, *n*=16; GSG1L, *n*=9; *P*<0.001; *t*-test). Scale bar, 100 pA and 2 s. (**d**) Left panel shows sample traces of normalized *I/V* curves recorded from glutamate-evoked somatic outside-out patch currents in control and GSG1L-overexpressing CA1 pyramidal neurons in cultured organotypic slices. Bar graph shows that there is no difference of the rectification index (control, *n*=9; GSG1L, *n*=13; *P*=0.57; *t*-test). (**e**) Expression of GSG1L-Myc strongly reduced surface GluA1 expression in cultured dissociated hippocampal neurons. (Left) Representative image of cultured hippocampal neurons (DIV17) expressing Myc-tagged GSG1L (red), and stained for surface GluA1 (green). Scale bar, 10 μm. The boxed area was shown in the middle. (Middle) Arrow heads indicate the surface expression of GluA1 (sGluA1) on a Myc-tagged GSG1L-negative dendrite and arrows indicate the surface expression of GluA1 on a Myc-tagged GSG1L positive dendrite. Scale bar, 5 μm. (Right) Scatter plot represents the distribution of each pair of cells analysed and shows that GluA1 surface expression on Myc-tagged GSG1L positive dendrites (GSG1L) was significantly reduced (*n*=6 independent cultures and 64 pairs of neighbouring neurons; *P*<0.001, *t*-test with *n*=64; *P*<0.01, *t*-test with *n*=6; *P*<0.01 with two-level nested ANOVA). Statistical significance is presented as ***P<0.001.

To carefully examine the role of GSG1L in negatively regulating AMPAR-mediated synaptic transmission, we performed molecular replacement assays in AMPAR triple conditional KO mice in which three genes encoding AMPAR subunits (GluA1, A2 and A3) are all conditional (*Gria1–3*^*f/f*^)^33^. Expression of Cre leads to a complete loss of AMPA EPSCs in neurons from *Gria1–3*^*f/f*^ mice^33^. Expression of Cre together with GluA1, rescued ∼75% of AMPAR synaptic transmission (Supplementary Fig. 4a) (refs 34, 35). Interestingly, expression of Cre with a construct that covalently linked GSG1L to the GluA1 C-tail in tandem (GluA1/GSG1L) only rescued ∼20% of AMPA EPSCs (Supplementary Fig. 4b and Fig. 2a). Importantly, when expressed in HEK cells, GluA1/GSG1L generated the same amount of whole-cell currents evoked by 1 mM glutamate as GluA1 and GSG1L expressed together (Supplementary Fig. 4d), indicating that these GluA1/GSG1L channels are properly assembled and appropriately respond to the agonist on the cell surface. In contrast, the plasmid in which the same strategy was used to link γ8 to GluA1 C-tail (GluA1/γ8) (ref. 24) rescued nearly 100% of AMPA EPSCs after being transfected together with Cre into hippocampal slice cultures prepared from *Gria1–3*^*f/f*^ mice (Supplementary Fig. 4c and Fig. 2b). It is worth noting that in neurons expressing Cre and GluA1/GSG1L, the ratio of kainate-evoked versus glutamate-evoked whole-cell currents was similar to nearby control neurons (Supplementary Fig. 4e), indicating that TARPs are still associated with GluA1/GSG1L fusion proteins^24^. Taken together, these data suggest that the association of GSG1L, but not γ8, with AMPARs prevents the receptor trafficking to synapses. Therefore, through a molecular replacement approach, we show that GSG1L negatively regulates AMPAR-mediated synaptic transmission.

### Overexpression of GSG1L reduced surface AMPARs in neurons

The strong reduction of AMPA EPSCs in neurons expressing GSG1L was accompanied by ∼80% decrease of AMPAR-mediated somatic outside-out patch currents (Fig. 2c), which has been used to measure AMPARs at somatic extrasynaptic membranes^10^,^11^,^33^,^36^. Current–voltage (*I/V*) relationship analysis in somatic outside-out patches demonstrated that there was no change of *I/V* relationship (Fig. 2d), indicating that the GluA2 content of somatic surface AMPARs was not altered^33^. In addition, in hippocampal primary neuronal cultures, neuronal surface immunolabelling of the GluA1 and GluA2 subunits, the major AMPAR subunits in hippocampal CA1 pyramidal neurons^33^,^37^, was strongly reduced (Fig. 2e and Supplementary Fig. 5), further supporting that GSG1L overexpression reduced the abundance of AMPARs at the neuronal surface.

### The Loop1 domain is important for the GSG1L effect on AMPARs

**Figure 3:**
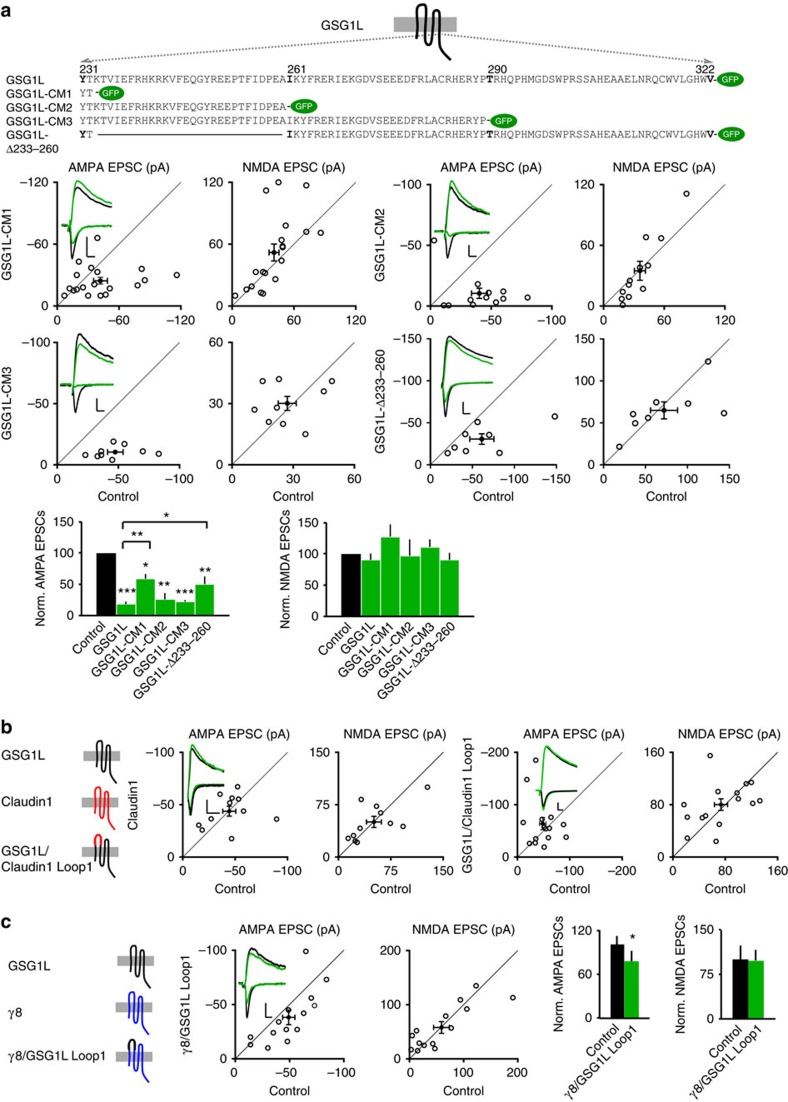
The GSG1L Loop1 domain is critical for the regulation of AMPA EPSCs. (**a**) Dual whole-cell recordings revealed that the first 28 amino acids in the GSG1L C-tail was involved in the negative regulation of AMPA EPSCs (GSG1L-CM1: AMPA, *n*=19; *P*<0.05; NMDA, *n*=19; *P*=0.10; GSG1L-CM2: AMPA, *n*=12; *P*<0.01; NMDA, *n*=12; *P*=0.81; GSG1L-CM3: AMPA, *n*=9; *P*<0.001; NMDA, *n*=9; *P*=0.57; GSG1L-Δ233–260: AMPA, *n*=8; *P*<0.01; NMDA, *n*=8; *P*=0.84; paired *t*-test). Scale bar, 20 pA and 20 ms. (**b**) The Loop1 domain was critical for GSG1L-dependent regulation of AMPAR EPSCs. In the GSG1L/Claudin1 Loop1 swap mutant, GSG1L Loop1 domain was replaced by the Loop1 domain from Claudin1. Neither Claudin1 (AMPA, *n*=11; *P*=0.93; NMDA, *n*=11; *P*=0.95; Scale bar, 50 pA and 20 ms) nor GSG1L/Claudin1 Loop1 (AMPA, *n*=14; *P*=0.31; NMDA, *n*=14; *P*=0.53; paired *t*-test. Scale bar, 30 pA and 20 ms) expression changed AMPA and NMDA EPSCs. (**c**) A γ8 chimeric mutant was made in which the γ8 Loop1 domain was swapped with that from GSG1L to generate γ8/GSG1L Loop1. Overexpression of γ8/GSG1L Loop1 in CA1 pyramidal neurons from cultured organotypic hippocampal slices led to a small but significant reduction of AMPA EPSCs (AMPA, *n*=14; *P*<0.05; NMDA, *n*=14; *P*=0.89; paired *t*-test). Scale bar, 20 pA and 20 ms. Statistical significance is presented as *P<0.05, **P<0.01 or ***P<0.001.

Both GSG1L and TARPs are tetraspanning membrane proteins (Fig. 1a) and belong to the Claudin superfamily^17^,^19^. Previous studies showed that the carboxyl-terminal (C-tail) domain of TARPs was important for the TARP-dependent regulation of AMPAR trafficking^38^,^39^,^40^,^41^. Thus, we were wondering if GSG1L shared a similar modular organization to modulate AMPAR-mediated synaptic transmission. Towards this end, we made a series of deletion mutants at the GSG1L C-tail with GFP fused to their carboxyl ends, biolistically expressed these mutants in hippocampal slice cultures, and performed dual recordings (Fig. 3a). Although overexpression of GSG1L-CM1 (C-tail mutant 1) that lacks the entire GSG1L C-tail inhibited AMPA EPSCs, its effect on AMPA EPSCs was significantly diminished (Fig. 3a). In contrast, both GSG1L-CM2 and GSG1L-CM3 strongly suppressed AMPA EPSCs, similar to wild-type (WT) GSG1L (Fig. 3a). These data suggest that the juxtamembrane region of 28 amino acids in the GSG1L C-tail may play a role in the negative regulation of AMPA EPSCs by GSG1L (Fig. 3a). Indeed, expression of a GSG1L mutant lacking the juxtamembrane region (GSG1L-Δ233–260) could significantly reduce AMPA EPSCs, but not NMDA EPSCs, although its effect on AMPA EPSCs was smaller as compared with full-length GSG1L (Fig. 3a). These data also suggest that there are other domain(s) in GSG1L that are important for the regulation of AMPA EPSCs. We therefore swapped the majority of GSG1L first extracellular Loop (Loop1) domain (amino acids 44–108) with that from Claudin1 (see Methods), a protein that did not change synaptic transmission after overexpression in neurons (Fig. 3b). The swap mutant (GSG1L/Claudin1 Loop1) completely abolished the inhibitory function of GSG1L in AMPA EPSCs, as overexpression of this mutant affected neither AMPA nor NMDA EPSCs (Fig. 3b), suggesting that the Loop1 is important for GSG1L function in the regulation of AMPA EPSCs. Importantly, all these mutants were expressed at similar levels in neurons (Supplementary Fig. 6a). Taken together, these results indicate that GSG1L regulates AMPA EPSCs through both the Loop1 domain- and C-tail-dependent mechanisms.

We also wondered whether the GSG1L Loop1 domain was sufficient for the inhibitory effect of GSG1L on AMPA EPSCs. To test this, we made a γ8 chimeric mutant in which the γ8 Loop1 domain was swapped with that from GSG1L (γ8/GSG1L Loop1, see Methods). We found that while overexpression of γ8 did not change AMPA EPSCs (Fig. 1c), expression of the γ8 swap mutant, γ8/GSG1L Loop1, led to a modest, but significant, reduction of AMPAR-mediated synaptic transmission (Fig. 3c). In contrast, expression of γ8/Claudin1 Loop1 mutant, in which γ8 Loop1 was swapped with that from Claudin1, did not significantly alter AMPA EPSCs (Supplementary Fig. 6b). Furthermore, we made another swap mutant in which the Claudin1 Loop1 domain was swapped with that from GSG1L (Claudin1/GSG1L Loop1, see Methods). Expression of Claudin1/GSG1L Loop1 also led to a modest, but significant, reduction of AMPA EPSCs (Supplementary Fig. 6c). In addition, all swap mutants were expressed at similar levels as full-length GSG1L (Supplementary Fig. 6a). These data indicate that GSG1L Loop1 is sufficient to confer γ8 or Claudin1 with an inhibitory function to regulate AMPA EPSCs.

### GSG1L KO enhances AMPA EPSCs and LTP at CA1 synapses

**Figure 4:**
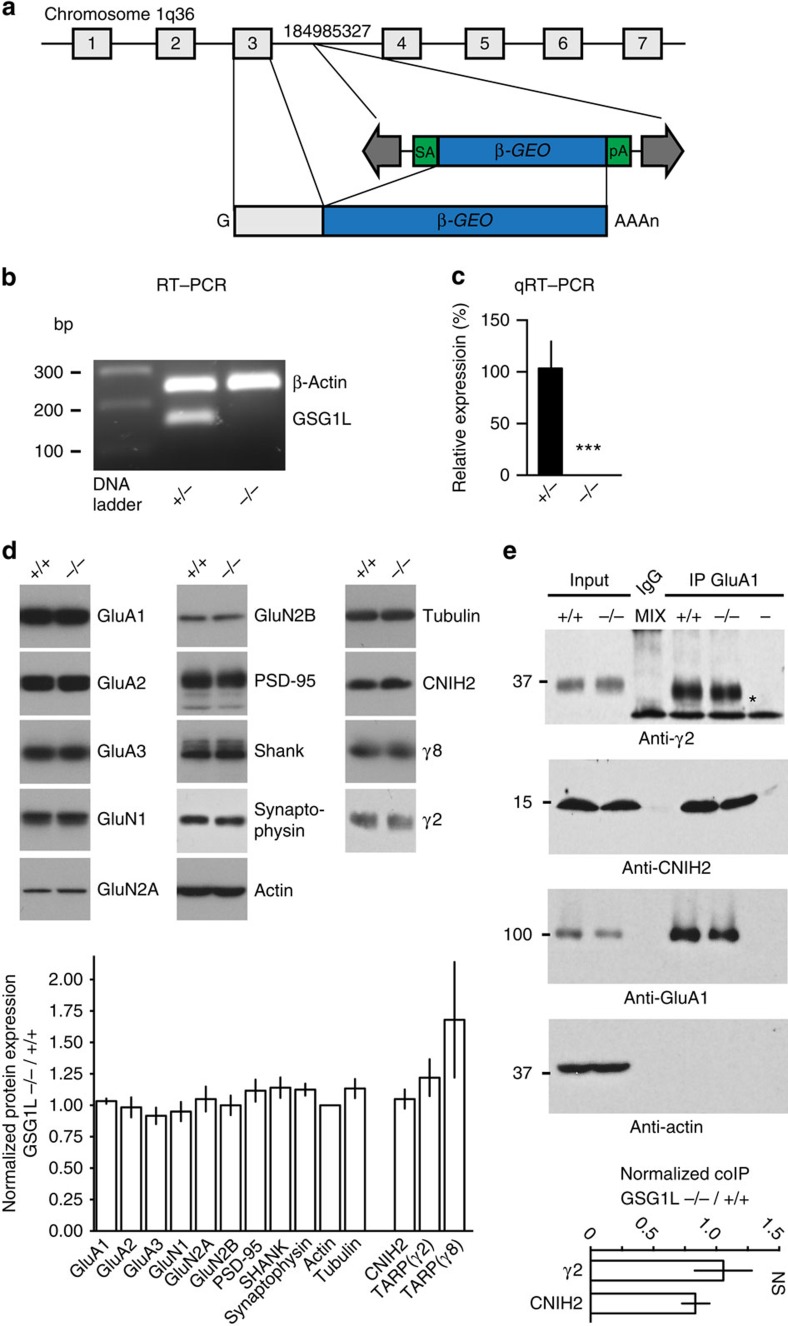
Characterization of *Gsg1l* knockout (KO) rats. (**a**) Schematic outline of a *Sleeping Beauty* gene trap in intron 3 of rat *Gsg1l*. SA, splice acceptor; pA, poly A. (**b**) Reverse transcription–PCR (RT–PCR) showed no detectable GSG1L mRNA in the hippocampal tissues of KO (−/−) rats. (**c**) Quantitative real-time RT–PCR (qRT–PCR) showed that homozygous GSG1L KO (−/−) rats lacked GSG1L mRNA in the hippocampal tissue as compared with the heterozygous rats (*P*<0.001, *t*-test). (**d**) Bar graph shows normalized expression of various synaptic proteins (GSG1L KO/WT). Six sets of hippocampi were dissected from P14–17 WT (+/+) and GSG1L KO (−/−) rats. Proteins prepared from crude synaptosomal membranes were immunoblotted, quantitated and normalized (*n*=6, *P*>0.05, One-way ANOVA test). Sample blots for each protein are shown above the bar graph. (**e**) Immunoprecipitation experiments in +/+ and −/− hippocampal lysates. Bar graph in the bottom shows that TARP γ2 and CNIH2 associated with GluA1 at comparable levels in +/+ and −/− hippocampal lysates (mix: mix of proteins from +/+ and −/− (50%/50%); ‘−', only lysate buffer without protein lysates; ‘*', TARP γ2-specific signal; *n*=3, *P*>0.05, *t*-test). Statistical significance is presented as ***P<0.001. NS, not significant.

**Figure 5:**
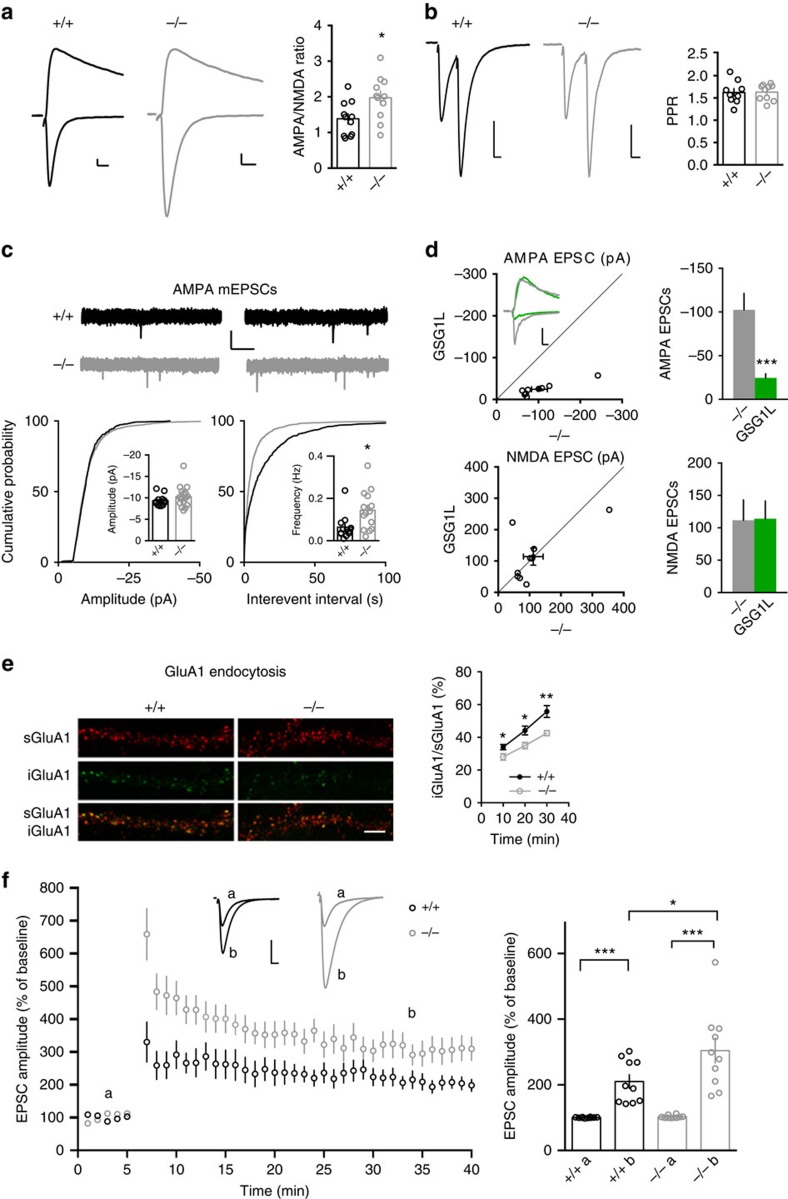
AMPA EPSCs and LTP were increased in CA1 pyramidal neurons in GSG1L KO rats. (**a**) Ratio of AMPAR- to NMDAR- synaptic transmission was significantly enhanced in KO rats (+/+, *n*=11; −/−, *n*=11; *P*<0.05; *t*-test). Scale bar, 20 pA and 20 ms. (**b**) There was no change of paired-pulse ratio (PPR) of AMPA EPSCs recorded in CA1 pyramidal neurons from GSG1L KO rats (+/+, *n*=9; −/−, *n*=10; *P*=0.91, *t*-test). Scale bar, 20 pA and 20 ms. (**c**) AMPAR-mediated mEPSC frequency in CA1 pyramidal neurons from p14–19 KO rats was significantly increased (amplitude: +/+, *n*=12; −/−, *n*=18; *P*=0.23; frequency: +/+, *n*=12; −/−, *n*=18; *P*<0.05; *t*-test; Kolmogorov–Smirnov test was used for cumulative distributions, *P*<0.001 for frequency and *P*>0.05 for amplitude). Scale bar, 10 pA and 1 s. (**d**) Overexpression of GSG1L in CA1 pyramidal neurons in cultured organotypic hippocampal slices prepared from GSG1L KO rats strongly reduced AMPAR-mediated synaptic transmission (AMPA, *n*=9; *P*<0.001; NMDA, *n*=9; *P*=0.94; paired *t*-test). Scale bar, 50 pA and 20 ms. (**e**) Reduced GluA1 endocytosis in GSG1L KO neurons (sGluA1, surface GluA1; iGluA1, internalized GluA1; 10 min, *P*<0.05; 20 min, *P*<0.05; 30 min, *P*<0.01; *n*=10 neurons in each time point). Sample images were taken from neurons at 10 min after chasing. Scale bar, 5 μm. (**f**) LTP at hippocampal CA1 synapses was significantly enhanced in GSG1L KOs (+/+, *n*=10, *P*<0.001; −/−, *n*=10, *P*<0.001; *n*=4 animals for each conditions; *P*<0.05 between +/+ and −/−; One-way ANOVA test). Bar graph shows the average normalized EPSCs before and after LTP induction. *P*<0.05 between +/+ and −/−. a, baseline EPSC (0–5 min); b, EPSC after LTP induction (30–35 min). Scale bar, 50 pA and 20 ms. Statistical significance is presented as *P<0.05, **P<0.01 or ***P<0.001.

To study the role of GSG1L in the regulation of synaptic transmission *in vivo*, we took advantage of a GSG1L KO rat line previously generated with the gene-trap approach (Fig. 4a–c)^42^. In the GSG1L KO rats, there was little change of the total expression of a number of neuronal proteins and there was no difference in co-IP of GluA1 with TARP γ2 and CNIH2 in protein lysates prepared from GSG1L KO hippocampi (Fig. 4d,e and Supplementary Figs 11 and 12). We reasoned that since GSG1L negatively modulates AMPA EPSCs, genetic deletion of GSG1L should enhance AMPAR-mediated synaptic transmission. We found that the surface immunolabelling of the GluA1 and GluA2 subunits in hippocampal neuronal cultures prepared from GSG1L KO was enhanced (Supplementary Fig. 7a,b), indicating increased expression of AMPAR subunits on the neuronal surface in the absence of GSG1L. In addition, AMPA/NMDA EPSCs ratios were increased in neurons from the hippocampal CA1 region in GSG1L KOs (Fig. 5a). There was no difference of PPR (Fig. 5b). We also analysed AMPA mEPSCs in CA1 pyramidal neurons from KO rats and found that mEPSC frequency was substantially increased (Fig. 5c), suggesting that there were more functional AMPAR-containing synapses in GSG1L-deficient CA1 pyramidal neurons. There was no significant difference for mEPSC amplitudes (Fig. 5c). Furthermore, expression of GSG1L in CA1 pyramidal neurons in organotypic slice cultures prepared from GSG1L KO rats strongly reduced AMPA EPSCs (Fig. 5d), suggesting that the synaptic abundance of AMPARs is sensitive to GSG1L-dependent mechanisms in the KO neurons. What is the mechanism underlying the enhanced surface expression of AMPARs in GSG1L KO neurons? We found that endocytosis of surface GluA1 (sGluA1) was reduced in neurons lacking GSG1L (Fig. 5e), although there was no change of recycling of internalized GluA1 (Supplementary Fig. 7c). In addition, endocytosis of sGluA1 was enhanced in neurons overexpressing GSG1L (Supplementary Fig. 7d). However, the strong reduction of sGluA1 led to little internalized GluA1 in GSG1L-overexpressing neurons, which prevented us from performing recycling assay in neurons overexpressing GSG1L. Taken together, these findings complement overexpression data and demonstrate that genetic deletion of GSG1L increases AMPAR-mediated synaptic transmission and GSG1L is involved in the regulation of AMPAR endocytosis.

We also examined LTP, which has been proposed to be a key cellular model for learning and memory^5^,^43^. We found that compared with WT rats, hippocampal LTP at the Schaffer-collateral pathway was significantly enhanced in GSG1L KO rats (Fig. 5f), indicating an important role of GSG1L in the regulation of LTP.

### GSG1L speeds up AMPAR channel kinetics in CA1 neurons

**Figure 6 f6:**
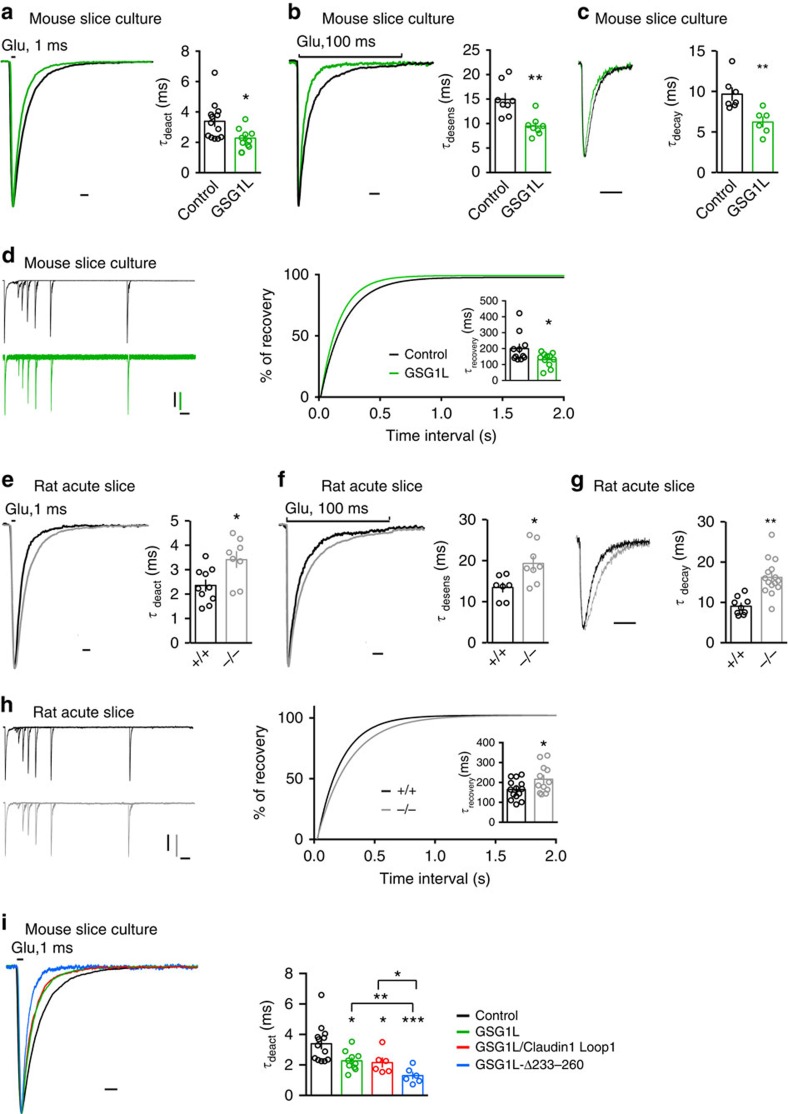
GSG1L modulates AMPAR kinetic properties in CA1 pyramidal neurons. (**a**–**c**) GSG1L overexpression in cultured organotypic hippocampal slices sped up AMPAR deactivation time constant (**a**, control, *n*=13; GSG1L, *n*=10; *P*<0.05; *t*-test; Scale bar, 2 ms), desensitization time constant (**b**, control, *n*=8; GSG1L, *n*=8; *P*<0.01; *t*-test; Scale bar, 10 ms) and mEPSC decay time constant (**c**, control, *n*=7; GSG1L, *n*=6; *P*<0.01; *t*-test; Scale bar, 20 ms) in CA1 pyramidal neurons. Peak-normalized sample traces are shown to the left for **a**–**c**. (**d**) GSG1L overexpression modestly accelerated the recovery of AMPARs from desensitization (control, *n*=11; GSG1L, *n*=11; *P*<0.05; *t*-test). Scale bar, 100 ms, 200 pA (black) and 20 pA (green). (**e**–**g**) In GSG1L KO CA1 pyramidal neurons, AMPAR deactivation time constant (**e**, +/+, *n*=10; −/−, *n*=8; *P*<0.05; *t*-test; Scale bar, 2 ms), desensitization time constant (**f**, +/+, *n*=7; −/−, *n*=8; *P*<0.05; *t*-test; Scale bar, 10 ms) and mEPSC decay time constant (**g**, +/+, *n*=9; −/−, *n*=15; *P*<0.01; *t*-test; Scale bar, 20 ms) were significantly slowed. Peak-normalized sample traces are shown to the left for **e**–**g**. It is worth noting that our overexpression experiments (**a**–**d**) were performed in mouse slice cultures and gene KO experiments (**e**–**h**) were performed in acute rat slices. Thus, the *τ* values for deactivation and desensitization in rat or mouse CA1 control neurons are different, but are consistent with published data^10^,^22^,^24^,^59^,^67^,^68^. (**h**) GSG1L KO modestly slowed the AMPAR recovery from desensitization (+/+, *n*=14; −/−, *n*=12; *P*<0.05; *t*-test). Scale bar, 100 ms, 100 pA (black) and 100 pA (grey). (**i**) Deletion of the juxtamembrane region in the GSG1L C terminus-accelerated AMPAR deactivation kinetics (control, *n*=13; GSG1L, *n*=10; GSG1L/Claudin1 Loop1, *n*=6; GSG1L-Δ233–260, *n*=6; One-way ANOVA test *P*<0.05, *P*<0.01, *P*<0.001). Peak-normalized sample traces are shown to the left. Scale bar, 2 ms. Statistical significance is presented as *P<0.05, **P<0.01 or ***P<0.001.

**Figure 7:**
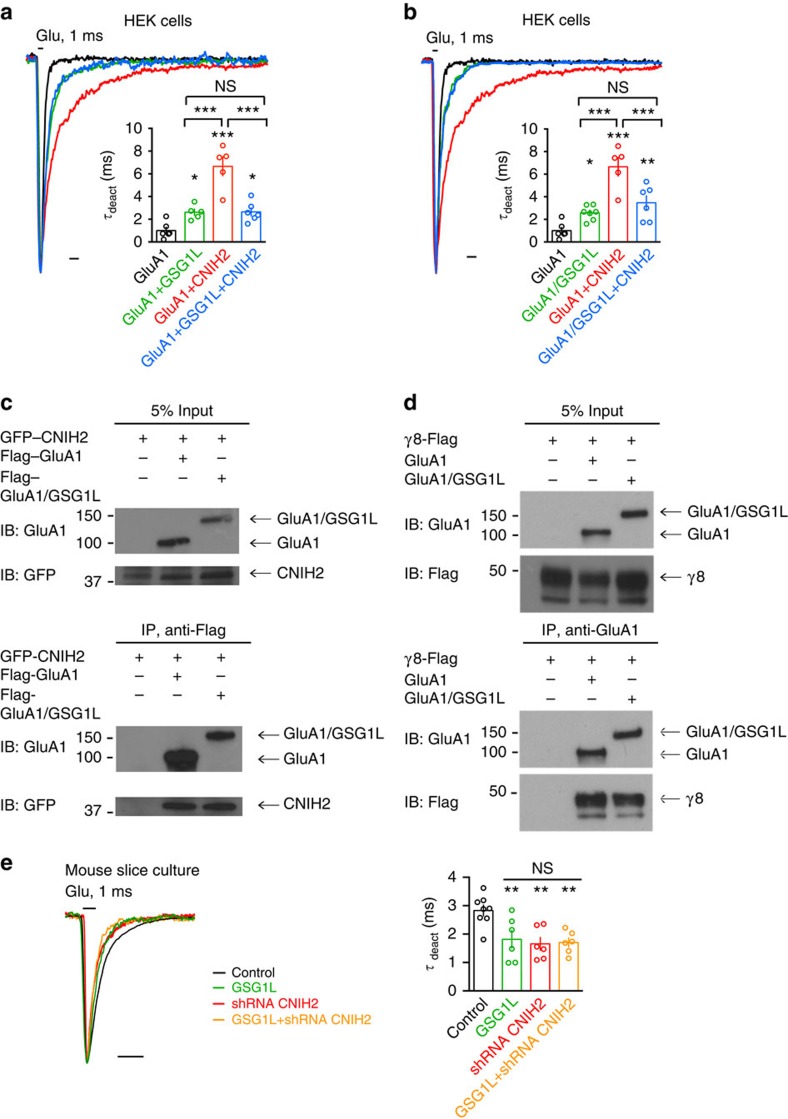
GSG1L inhibits CNIH2 effect on AMPAR deactivation kinetics. (**a**,**b**) Bar graph shows weighted time constants of deactivation of GluA1 homomers (**a**, GluA1, *n*=6; GluA1+GSG1L, *n*=5; GluA1+CNIH2, *n*=5; GluA1+CNIH2+GSG1L, *n*=6; *P*<0.01) or GluA1/GSG1L homomers (**b**, GluA1, *n*=6; GluA1/GSG1L, *n*=7; GluA1+CNIH2, *n*=5; GluA1/GSG1L+CNIH2, *n*=6; *P*<0.01) in HEK cells; One-way ANOVA test; *P* value between individual groups was derived from multiple comparison of ANOVA test as shown in the figure: *P*<0.05, *P*<0.01, *P*<0.001). Peak-normalized sample traces are shown above the bar graph. Scale bar, 2 ms. (**c**,**d**) Co-IP of GluA1 or GluA1/GSG1L fusion protein with CNIH2 (**c**) or γ8 (**d**) in HEK cells showed that both GluA1 and GluA1/GSG1L fusion protein were co-immunoprecipitated with CNIH2 (**c**) or γ8 (**d**). About 5% of the lysate used for IP (input) was also probed with indicated antibodies to confirm protein expression (top). (**e**) CNIH2 knockdown occluded the effect of GSG1L overexpression on AMPAR deactivation kinetics in CA1 pyramidal neurons (control, *n*=8; GSG1L, *n*=6; shRNA CNIH2, *n*=6; GSG1L+shRNA CNIH2, *n*=6; *P*<0.01 for One-way ANOVA test; *P* value between individual groups was derived from multiple comparison of ANOVA test as shown in figure, *P*<0.01). Peak-normalized sample traces are shown in the left panel. Please note that AMPAR deactivation kinetics were slightly faster in older cultures shown here than in younger cultures shown in Fig. 6a, although the difference was not significant (two-tailed *t*-test, *P*=0.3 between control *τ* numbers in Fig. 6a and 7e, *P*=0.22 between GSG1L *τ* numbers in Fig. 6a and 7e). We performed these experiments in Fig. 7e in older cultures because we had to wait for enough time for shRNA knockdown of CNIH2 (over a week), and thus GSG1L was also overexpressed for a longer time in this experiment. Scale bar, 2 ms. Statistical significance is presented as *P<0.05, **P<0.01 or ***P<0.001. NS, not significant.

Previously, it has been shown that GSG1L modulates AMPAR gating kinetics in heterologous cells^8^,^9^. Specifically, GSG1L slowed AMPAR deactivation, desensitization and the recovery from desensitization in HEK cells or in *Xenopus laevis* oocytes^8^,^9^. We also found that GSG1L slowed AMPAR deactivation kinetics in HEK cells[Fig f6] (Fig. 7a). Thus, based on data from heterologous cells, one would predict that overexpression of GSG1L in neurons would also slow AMPAR kinetics or have no effect if GSG1L expression was saturated in neurons, as in the case for the TARP γ8 (ref. 24). Surprisingly, overexpression of GSG1L in CA1 pyramidal neurons significantly sped up both deactivation and desensitization kinetics of AMPARs measured in somatic outside-out patches (Fig. 6a,b). mEPSC analysis from neurons overexpressing GSG1L indicated that mEPSC decay kinetics was faster than that in control neurons (Fig. 6c). In addition, overexpression of GSG1L in CA1 neurons modestly sped up the receptor recovery from desensitization (Fig. 6d). Furthermore, biophysical analysis in CA1 pyramidal neurons from KO rats showed that genetic deletion of GSG1L slowed the deactivation and desensitization kinetics of AMPARs from outside-out patches (Fig. 6e,f) and the decay constant of synaptic AMPARs (Fig. 6g). The recovery of AMPARs from desensitization in CA1 neurons was also slightly slower in GSG1L KOs (Fig. 6h). Taken together, these data suggest that although GSG1L slows kinetics of recombinant AMPARs in isolation in heterologous cells, it speeds up kinetics of native AMPARs in hippocampal CA1 pyramidal neurons.

We also examined the role of the GSG1L juxtamembrane region and extracellular Loop1 domain in the regulation of AMPAR deactivation kinetics in neurons. We found that similar to full-length GSG1L, GSG1L/Claudin1 Loop1 accelerated AMPAR deactivation kinetics in CA1 pyramidal neurons (Fig. 6i). Surprisingly, GSG1L-Δ233–260, a GSG1L mutant lacking the juxtamembrane region in the C-tail, caused an even greater speeding of deactivation kinetics (Fig. 6i). These data suggest that these domains play differential roles in the regulation of AMPA EPSCs (Fig. 3) and in the modulation of AMPAR kinetic properties (Fig. 6i).

### GSG1L inhibits the CNIH2 effect on AMPAR deactivation

Several factors, including AMPAR subunit composition and subunit alternative splicing isoforms, differential posttranslational modifications of the receptors or multiple AMPAR auxiliary subunits expressed in neurons, could account for the kinetic differences between recombinant AMPARs expressed in heterologous cells and endogenous AMPARs. Accumulating evidence has shown that neuronal AMPAR complexes contain many auxiliary subunits that may impose complex effects on AMPAR function^7^,^8^,^9^,^10^,^11^,^13^,^15^,^44^. Indeed, in hippocampal neurons, TARP γ8 can functionally interact with CNIHs or CKAMP44 to regulate AMPAR kinetics^10^,^13^,^15^,^44^. Thus, it is possible that in neurons the presence of multiple auxiliary subunits, such as TARPs, CNIHs, CKAMP44 and GSG1L, may alter their functions in the regulation of AMPAR gating kinetics. Indeed, a previous study in *Xenopus* oocytes has shown that while GSG1L did not modify TARP γ2's function on AMPARs, it could reverse the CNIH2 effect on the time constants of deactivation and desensitization^8^. In agreement with this study^8^, our experiments in HEK cells showed that while CNIH2 profoundly slowed GluA1 homomer deactivation kinetics, co-expression of GSG1L and CNIH2 with GluA1 prevented CNIH2-induced slowing (Fig. 7a, Supplementary Figs 8a and 15a), suggesting that GSG1L is capable of modifying the effect of CNIH2 on AMPAR gating.

A caveat of co-expression of GluA1, GSG1L and CNIH2 in HEK cells is that it might generate AMPARs with variable stoichiometry with different auxiliary subunits. Towards this end, we took advantage of the GluA1/GSG1L fusion construct that we have used in the molecular replacement experiments (Fig. 2a, Supplementary Figs 4, 8b and 15b). We found that in HEK cells, deactivation kinetics of GluA1/GSG1L were similar to GluA1 co-expressed with GSG1L (Fig. 7a,b). When GluA1/GSG1L and CNIH2 were co-expressed, CNIH2 was no longer capable of exerting its profound effect on AMPAR deactivation kinetics (Fig. 7b). Interestingly, co-IP experiments in HEK cells showed that both GluA1 and GluA1/GSG1L could effectively pull down CNIH2, demonstrating that GluA1/GSG1L complexes are capable of interacting with CNIH2 (Fig. 7c and Supplementary Fig. 13c). In addition, both GluA1 and GluA1/GSG1L could co-immunoprecipitate with γ8 (Fig. 7d and Supplementary Figs 8c,13d and 15c). These data indicate that while GSG1L can reverse CNIH2 effects on AMPARs, it does not prevent CNIH2 from binding to AMPARs.

Finally, we reasoned that if GSG1L acts on CNIH2 to regulate AMPAR deactivation kinetics, then GSG1L might not have an effect on AMPAR deactivation in neurons lacking CNIH2. In agreement with a previous report^10^, we found that expression of a CNIH2 short hairpin RNA (shRNA) construct that effectively knocked down CNIH2 (ref. 10), but not a control scramble shRNA construct, in hippocampal CA1 neurons sped up AMPAR deactivation kinetics (Fig. 7e and Supplementary Fig. 9). Co-expression of GSG1L with the CNIH2 shRNA construct had no further effect on AMPAR deactivation kinetics (Fig. 7e). Indeed, AMPAR deactivation kinetics are indistinguishable in neurons with GSG1L overexpression, with CNIH2 knockdown, or with both GSG1L overexpression and CNIH2 knockdown (Fig. 7e), suggesting that loss of CNIH2 occludes the effect of GSG1L on AMPAR deactivation kinetics.

### GSG1L is important for non-spatial object recognition memory

**Figure 8:**
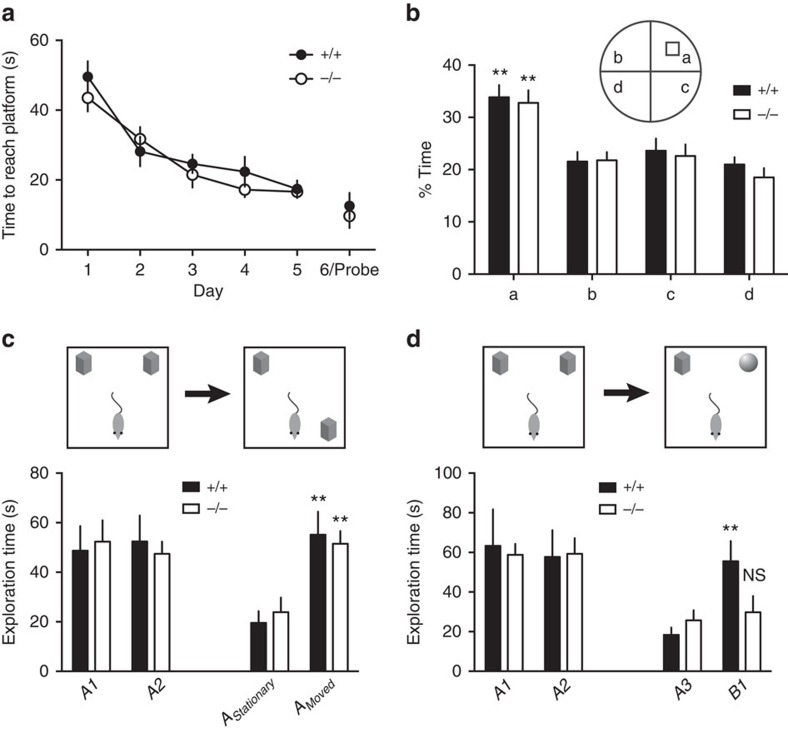
GSG1L KO rats have deficits in a non-spatial object recognition memory test. (**a**) Morris water maze testing showed that there was no difference between 3–5 month-old male +/+ (WT, *n*=18) and −/− (KO, *n*=19) rats in latency to locate the hidden platform during acquisition days 1–5 (two-way ANOVA with the between-subjects factor of Strain and the within-subjects factor of Day; *P*>0.05) or during the probe trial (day 6) in time to first enter the platform zone (*t*-test between strains; *P*>0.05). (**b**) The rats were tested in a probe trial for spatial memory retention after a 24-h delay following 5-day acquisition training. Both +/+ and −/− spent significantly more time in the target quadrant (a) as compared with quadrants (b, c and d) (+/+, *n*=10; −/−, *n*=19; two-way ANOVA with the between-subjects factor of Strain and the within-subjects factor of Quadrant followed by Tukey's multiple comparison tests; *P*<0.01). However, there was no difference between the two genotypes in time spent in the target quadrant (*t*-test between strains; *P*>0.05). (**c**) The spatial object recognition memory test showed that spatial object memory was normal in KO rats (+/+, *n*=9; −/−, *n*=9; *P*<0.05 for both genotypes; mixed-model ANOVA with the between-subjects factor of Strain and the within-subjects factors of Test and Object followed by Tukey's *post hoc* tests). Both genotypes showed a similar significant increase of their exploration time with the moved object in the novel spatial position. (**d**) The −/− rats had a deficit in the non-spatial object recognition memory test. While +/+ rats showed a strong preference towards the novel object (object B1), the −/− rats did not show a difference in exploration time between familiar (object A3) and novel (object B1) objects (+/+, *n*=9, *P*<0.01; −/−, *n*=9; *P*=0.68; mixed-model ANOVA with the between-subjects factor of Strain and the within-subjects factors of Test and Object followed by Tukey's *post hoc* tests). Statistical significance is presented as **P<0.01. NS, not significant.

AMPAR trafficking and function have been implicated in animal behaviour and cognition^1^,^45^. The important effects that GSG1L exerts on AMPAR synaptic transmission in hippocampal neurons prompted us to investigate the mutant rats for hippocampus-dependent cognition tests. We first tested the mutant rats in the standard Morris water maze task with a hidden platform, which is widely used for testing spatial learning and declarative memory that are believed to depend on hippocampal function^46^. Both WT and their littermate KO rats spent a similar amount of time to locate the hidden platform to escape the water, and both genotypes improved their escape latencies during the training trials (Fig. 8a and Supplementary Fig. 10a–c), suggesting normal spatial learning in the KO rats. We then performed the probe trial test in which the platform was removed, and measured the percentage of time spent in the quadrant where the platform was previously located. Interestingly, both genotypes of rats spent a similar amount of time in the target quadrant searching for the platform (Fig. 8b), indicating that the spatial memory to remember the platform location in the quadrant was comparable between the two genotypes.

Object recognition memory is another type of declarative memory that critically depends on hippocampal function^47^,^48^. There are two types of object recognition behavioural tasks that measure spatial and non-spatial memory, respectively. In the spatial object recognition experiments, the recognition of the novelty of the object position in space, but not the object itself, is tested. On the contrary, the non-spatial object recognition task tests the memory that recognizes the novelty of the object itself. In either spatial (Fig. 8c) or non-spatial (Fig. 8d) object recognition tasks, both WT and GSG1L KO rats spent a similar amount of time with the two objects in an open-field box during the familiarization session (Fig. 8c,d, left, and Supplementary Fig. 10d,e). In the probe trial of the spatial object recognition experiment in which one of the objects was relocated to a novel position in space, we found that both genotypes exhibited a strong preference for the object in the novel space location (Fig. 8c, right). Thus, the memory for spatial object recognition did not differ between the two groups. In contrast, in the probe trial of the non-spatial object recognition experiment in which one of the objects was replaced by a novel object, while WT rats showed a profound preference for the novel object, the preference was completely abolished in the mutant rats (Fig. 8d, right). Taken together, these data demonstrate that physiological function of neuronal GSG1L is important for non-spatial novel object recognition memory.

## Discussion

In mammalian neurons, the vast majority of AMPAR auxiliary subunits (that is, TARPs, CNIHs and CKAMP44) discovered thus far have been shown to positively regulate AMPAR forward trafficking to the neuronal surface and synapses^6^,^7^,^10^,^11^,^13^,^15^,^20^,^21^,^38^,^44^,^49^,^50^,^51^,^52^,^53^. In this study, our data indicate that GSG1L plays an opposite role to TARPs/CNIHs/CKAMP44 in the regulation of AMPAR-mediated synaptic transmission. Indeed, overexpression of GSG1L led to an ∼80% reduction of AMPA EPSCs and somatic extrasynaptic AMPAR-mediated current amplitudes in CA1 pyramidal neurons, indicating that GSG1L suppresses AMPAR delivery to the cell surface and synapses. Consistent with this negative effect, AMPA EPSCs were increased in GSG1L KO CA1 pyramidal neurons. In addition, GSG1L regulates AMPAR endocytosis. In neurons overexpressing GSG1L, AMPAR endocytosis was enhanced, and conversely, in GSG1L KO neurons, the receptor endocytosis was reduced. In addition, GSG1L sped up the AMPAR deactivation and desensitization kinetics, and modestly accelerated the recovery of the receptors from desensitization in CA1 pyramidal neurons. Thus, GSG1L suppresses AMPAR abundance at synapses and renders faster glutamatergic synaptic transmission between neurons.

Both TARPs and GSG1L are tetraspanning membrane proteins and belong to the Claudin protein superfamily^19^. However, they differ in their dependence on protein domains in the regulation of AMPAR-mediated synaptic transmission. Previous work indicates that TARPs primarily utilize intracellular domains in the regulation of AMPAR synaptic targeting^6^,^21^,^38^,^39^,^40^. In contrast, although the GSG1L intracellular C-tail is involved in the regulation of AMPA EPSCs, the extracellular Loop1 domain is critical for its suppression of AMPAR-mediated synaptic transmission. GSG1L Loop1 is also sufficient for GSG1L's effect on AMPARs, as the TARP γ8 or Claudin1 chimera with the Loop1 domains replaced by that from GSG1L converted the γ8 or Claudin1 into negative regulators for AMPAR-mediated synaptic transmission. Interestingly, while the majority of GSG1L Loop1 (residues 44–108) is not critical for the regulation of AMPAR deactivation kinetics, the juxtamembrane region in its C-tail is important. Structural mechanisms underlying the regulation of AMPAR kinetics by the GSG1L C-tail remain unclear. It is worth mentioning that the AMPAR GluA1 C terminus has been shown to play an important role in modulating AMPAR gating^54^. Thus, it is possible that the juxtamembrane region in the GSG1L C-tail may regulate AMPAR gating through functional interaction with the GluA1 C terminus.

In addition, GSG1L and TARPs/CNIHs differ from each other in their regulation of AMPAR channel kinetic properties in neurons. In heterologous cells and in hippocampal pyramidal neurons, both TARPs and CNIHs increase time constants of AMPAR deactivation and desensitization kinetics^7^,^10^,^13^,^17^,^22^,^23^,^24^,^25^,^26^,^27^,^29^,^32^. In contrast, our data show that GSG1L plays an opposite role to TARPs/CNIHs in the regulation of AMPAR channel gating in hippocampal CA1 pyramidal neurons. Indeed, overexpression of GSG1L sped up deactivation and desensitization kinetics, and conversely, deactivation and desensitization were slowed in GSG1L KO CA1 pyramidal neurons. These data demonstrate that while TARPs/CNIHs slow the receptor kinetics, GSG1L speeds the rate of AMPAR deactivation and desensitization in hippocampal CA1 neurons.

Intriguingly, our data show that GSG1L differentially modulates gating kinetics of AMPARs in heterologous cells and in neurons. In heterologous cells, GSG1L modestly slowed AMPAR deactivation and desensitization kinetics, and profoundly decelerated the receptor recovery from desensitization^8^,^9^. In contrast, overexpression of GSG1L in CA1 pyramidal neurons decreased the time constants of AMPAR deactivation and desensitization, and also slightly accelerated the recovery from desensitization. How does GSG1L function differently in heterologous cells and in hippocampal CA1 pyramidal neurons? It is possible that complex AMPAR subunit composition/stoichiometry in neurons or neuron-specific posttranslational modifications of GSG1L and/or AMPARs may confer GSG1L with different capacities to regulate AMPARs in hippocampal pyramidal neurons as compared in heterologous cells. It is also possible that combinatorial expression of multiple AMPAR auxiliary subunits in neurons may induce complex effect of auxiliary subunits on AMPAR function. Indeed, GSG1L inhibited CNIH2-induced slowing of AMPARs in HEK cells, and thus resulted in faster deactivation kinetics, as CNIHs profoundly slowed AMPAR kinetics (Fig. 7) (refs 7, 8, 13, 22, 26, 29). In addition, we found that knockdown of CNIH2 in hippocampal CA1 neurons occluded the effect of overexpression of GSG1L on AMPAR deactivation kinetics. It is worth noting that GSG1L appears to specifically regulate CNIHs' effect on AMPARs, as it has been reported that GSG1L does not modulate TARPs' function in AMPAR gating^8^ and our data also showed normal kainate efficacy of GluA1/GSG1L fusion protein in neurons (Supplementary Fig. 4e). Similar mechanisms have recently been described for the functional interaction between CNIH2/3 and AMPARs by TARPs^10^,^13^,^31^,^44^ or between γ8 and CKAMP44 (ref. 15). It should be emphasized that it is unlikely that CNIH2 underlies all unique properties of GSG1L in neurons. For example, overexpression of GSG1L in neurons slightly sped AMPAR recovery from desensitization (Fig. 6d). However, CNIHs have been shown to have no effect on AMPAR recovery from desensitization^7^. Thus, additional mechanisms exist in neurons for the function of GSG1L in the regulation of AMPAR properties.

In addition, it has been reported that GSG1L increased GluA2 trafficking to the plasma membrane in HEK cells^9^. In contrast, we observed profound reductions of AMPAR-mediated synaptic transmission, AMPAR-mediated outside-out patch currents and the sGluA1 immunolabelling in neurons overexpressing GSG1L. Whole-cell currents in HEK cells expressing GluA1 and GSG1L were also significantly diminished as compared with cells expressing GluA1 on its own. It is unclear what accounts for the discrepancies, although different experimental preparations (HEK cells versus neurons in our study), different technical approaches in HEK cells (immunolabelling versus electrophysiology in our study) and different AMPAR subunits used in HEK cells (GluA2 versus GluA1 in our study) may explain the differences.

GSG1L also functions differently from two other transmembrane proteins, SynDIG1 and CKAMP44, that bind AMPARs^11^,^12^,^14^,^15^, in the regulation of AMPAR-mediated synaptic transmission. Although SynDIG1 does not appear to directly regulate AMPAR trafficking and kinetic properties^14^, CKAMP44 acts as an auxiliary subunit for AMPARs^11^,^15^. Overexpression and gene KO experiments have shown that CKAMP44 is not necessary for AMPAR forward trafficking to synapses in CA1 pyramidal neurons^11^,^15^. In contrast, GSG1L plays an important role in the regulation of AMPA EPSCs in the CA1 region. For the receptor channel gating, GSG1L and CKAMP44 exert overlapping but also distinct regulatory effects. CKAMP44 slows AMPAR deactivation kinetics, but speeds up desensitization kinetics^11^,^15^. In contrast, GSG1L decreases the time constants of both deactivation and desensitization. In addition, while CKAMP44 slows the recovery of AMPARs from desensitization^11^,^15^, GSG1L in neurons modestly accelerates this process.

As integral constituents of native AMPAR complexes in the brain, AMPAR auxiliary subunits are critically involved in synaptic plasticity. Indeed, hippocampal LTP at Schaffer-collateral-CA1 synapses was strongly impaired in TARP γ8 or CNIH2/3 KO mice^10^,^15^,^20^. On the contrary, LTP was significantly enhanced at CA1 synapses in GSG1L KOs. It is possible that in CA1 pyramidal neurons from GSG1L KO animals, reduced AMPAR endocytosis and the removal of GSG1L inhibitory effects on AMPAR trafficking facilitates AMPAR delivery and stabilization at synapses during LTP, which may underlie the enhanced LTP observed in GSG1L KOs. Alternatively, the differential regulation of the pool size of extrasynaptic AMPARs by different auxiliary subunits may be a key for their roles in LTP. It has been demonstrated that the size of the extrasynaptic reserve pool of AMPARs is critical for LTP expression^35^. Consistent with this notion, somatic extrasynaptic AMPAR-mediated currents in hippocampal CA1 pyramidal neurons are strongly reduced in TARP γ8 or CNIH2/3 KO mice^10^,^20^, but are significantly enhanced in GSG1L KO rats (data not shown).

Negative regulation of AMPAR trafficking by auxiliary subunits appears to be an evolutionarily conserved mechanism. Indeed, in *Caenorhabditis elegans*, CNIH homologue *cni-1* negatively controls AMPAR forward trafficking^55^. Although in mammalian neurons CNIHs are important for positively regulating AMPAR trafficking^7^,^10^, our data indicate that in mammalian neurons GSG1L assumes the role of an inhibitory auxiliary subunit for AMPARs, allowing a balanced control of abundance and function of AMPARs at synapses.

The regulation of AMPAR-mediated synaptic transmission is critical for animal behaviour and cognition^1^,^45^. As integral components of native AMPAR complexes in the brain, AMPAR auxiliary subunits exert delicate control on almost every aspect of AMPAR trafficking and function. However, little is known about the role of such fine regulatory mechanisms conferred by AMPAR auxiliary subunits in animal cognition in mammals. Although many behavioural abnormalities have been reported in stargazer mutant mice in which TARP γ2 is mutated^56^, the role of other auxiliary subunits, such as TARP γ8, CNIH2 and CKAMP44 that are enriched in hippocampal neurons, in animal behaviour remains largely unknown. Our data now show that the physiological function of GSG1L is critical for hippocampus-dependent non-spatial object memory. Interestingly, although there are broad changes in AMPAR synaptic transmission in GSG1L KOs, the performance of mutant animals in the standard Morris water maze is not impaired. This is reminiscent of behavioural phenotypes of GluA1 KO mice, which exhibit normal Morris water maze learning and memory^57^, but have strong deficits in AMPAR synaptic transmission at CA1 synapses^58^,^59^. Thus, it is plausible that spatial reference memory as revealed by the Morris water maze test is less sensitive to changes of AMPAR synaptic transmission. In contrast, other memories, such as object memory, require physiological functions of GSG1L.

## Methods

### Production of *Gsg1l* mutant rats

*Sleeping Beauty β-Geo* trap transposons^60^ were used to select mutant rat spermatogonial libraries *in vitro*^42^. Spermatogonial lines used to select mutant spermatogonial libraries were derived from Sprague Dawley rats (Hsd: Sprague Dawley, Envigo, Inc.). Spermatogonia comprising a selected library were then transplanted into rat testes for production of mutant spermatozoa^61^. Recipient males were bred with WT females to produce a random panel of donor cell-derived mutant rat strains enriched with gene traps in protein-coding genes^42^. Genomic sites of transposon integration were defined in the newly generated mutant rats by splinkerette PCR^42^ and sequence analysis alignment on genome build RGSC v3.4 (Rn4). One rat harboured a *Sleeping Beauty* gene trap in the third intron of *Gsg1l*, and this animal was subsequently outcrossed to WT Harlan Sprague Dawley stock to generate the colony of *Gsg1l* mutant rats [RRID: RGD_1562278 (gt184985327fkh)] used in this study*. Gsg1l* gene-specific PCR primers near *Sleeping Beauty* integration sites were used in combination with transposon-specific primers to genotype progeny (forward primer: 5′- ACGTTGTAGTGACCCCAAGC -3′, and reverse primer: 5′- TGCACGCATACTCACAATGA -3′). Rat protocols to generate the *Gsg1l* strain were approved by the Institutional Animal Care and Use Committee (IACUC) at UT Southwestern Medical Center in Dallas, as certified by the Association for Assessment and Accreditation of Laboratory Animal Care International (AALAC). Rat housing, breeding and handling protocols were approved by NINDS ACUC at NIH. Rats of both sexes at the age of p13–p19 were used for acute slice electrophysiology, rats of both sexes at the age of p1 were used for dissociated hippocampal cultures, rats of both sexes at the age of p6–p8 were used for organotypic hippocampal slice cultures. Male rats at the age of 3–5 months were used in behavioural assays.

### Plasmids

Mouse pCMV6-GSG1L–GFP fusion protein plasmid was purchased from ORIGENE (MG214180). pCAG-GSG1L-IRES–GFP plasmid was generated by inserting *GSG1L*-coding sequence to pCAGGS-IRES–GFP vector. C-terminal Myc-tagged GSG1L construct was generated by PCR amplifying GSG1L complementary DNA (cDNA) from GSG1L–GFP before ligating the amplicon in pcDNA3.0 (Invitrogen). pEGFP-Claudin1–GFP plasmid was a gift from Dr Tianyi Wang at the University of Pittsburgh. GSG1L C-tail deletion mutants was cloned by standard PCR and inserted into pCMV6–GFP vector with GFP tag in the C-termini. Transmembrane domain helices were predicted by HMMTOP (http://www.enzim.hu/hmmtop)^62^,^63^ for GSG1L swap mutant construction. Overlapping PCR was used to generate GSG1L/Claudin1 Loop1 swap mutant (pCMV6-GSG1L/Claudin1 Loop1–GFP) in which amino acids from 44–108 of the first extracellular loop of GSG1L was replaced by the amino acids from 28–81 of the first extracellular loop of Claudin1. Claudin1/GSG1L–GFP Loop1 swap mutant (pCMV6-Claudin1/GSG1L Loop1–GFP) was generated by replacing amino acids 43–66 of Claudin1 with amino acids 29–123 of GSG1L. All mutants have a GFP tag in the C-termini. Amino acids 51–119 of γ8 were replaced with amino acids 29–123 of GSG1L or amino acids 28–81 of Claudin1 in γ8/GSG1L(pCMV-γ8/GSG1 L Loop1–GFP) and γ8/Claudin1 (pCMV-γ8/Claudin1 Loop1–GFP), respectively. γ8/GSG1L and γ8/Claudin1 coding sequences were synthesized by BIO BASIC, INC. (Amherst, USA). pIRES2-GluA1 (flip), pIRES2-GluA1/γ8-IRES–GFP fusion construct and pIRES2-γ8-IRES–GFP were a gift from Dr Nicoll Roger's lab at UCSF^24^. Flag–CNIH2 and GFP–CNIH2 plasmids were described before^10^,^22^. pIRES2-GluA1/GSG1L-IRES–GFP fusion construct was made with a similar strategy as previously described^24^. Briefly, standard PCR was carried out with GluA1 template by using: forward primer, 5′- ACCTCGAGGCCACCATGCCGTACATCTTTGCCTTTTTCTGC -3′; reverse primer, 5′- TGTAATTCCTGTTGCTGTTGCTGTTGCTGTTGCTGTTGCAATCCTGTGGC TCCCAAGGG -3′. The product was inserted into pIRES2–GFP vector with XhoI and EcoRI sites. PCR product of GSG1L was generated by using forward primer: 5′- ACGAATTCATGAAGACGAGCCGCCGCGGC -3′ and reverse primer: 5′- GTGTCGACTCACACCCAGTGCCCCAGGACCCA -3′, and inserted into the GluA1 containing pIRES2–GFP vector with EcoRI and SalI sites. Thus, GSG1L was fused into C terminus of GluA1 with a short linker sequence (Q)10EFAT. The CNIH2 shRNA (target sequence: 5′- GATGCGGTCTCTATCATGA -3′) has been characterized before^10^. All plasmids were confirmed by DNA sequencing.

### Co-IP and western blot

HEK293T cells expressing indicated plasmids, were homogenized using micropestles in lysis buffer containing 25 mM Tris pH 7.5, 1% Triton X-100, 150 mM NaCl, 5% glycerol, 1 mM EDTA and protease inhibitors (Roche). Equal amounts of total lysates were pre-cleared with Protein G PLUS-Agarose beads (Santa Cruz SC-2002), and then incubated with anti-Flag M2 monoclonal antibody (Sigma F3165) and Protein G PLUS-Agarose beads overnight at 4 °C. Beads were washed six times with lysis buffer and boiled in equal amount of 2 × Laemmli Sample Buffer (Bio-Rad 161–0737) containing 5% of β-mercaptoethanol (BME) (Fisher Scientific BP176100). Immunoprecipitates were resolved by SDS–PAGE (Bio-Rad 4561083) and immunoblotted with anti-Myc (rabbit, 1:1,000, Cell Signaling Technology 2278S) and anti-Flag (rabbit, 1:1,000, Sigma F2555) monoclonal antibodies. For co-IP experiments, Flag–GluA1, Flag–GluA1/GSG1L and GFP–CNIH2 constructs were used. Anti-Flag M2 affinity gel (30 μl per lysate, Sigma A2220) was used for immunoprecipitating GluA1 and mouse anti-GluA1 (Millipore MAB2263) was used to detect GluA1, and rabbit anti–GFP (Invitrogen A-11122) was used to detect GFP–CNIH2 fusion protein. For co-IP experiments of haemagglutanin (HA)–GluA1 and HA–GluK1 with Flag–GSG1L or γ8-Flag, HA–GluA1 and HA–GluA1 were probed with a rabbit anti-HA antibody (Santa Cruz, S-805). For the expression level experiments, HEK293T cells were transfected with Flag–GluA1, Flag–CNIH2 and GSG1L-Myc at the ratio of 8:2:1, or with GluA1/GSG1L and Flag–CNIH2 at the ratio of 4:1 (the same ratio as in the kinetics experiments). Empty pcDNA3.0 vector were added to balance the amount of DNA in the transfection reagent (Effectene Transfection Reagent, Qiagen, 301425). The blot images were analysed by gel analysis function in ImageJ software (NIH). The relative density was determined by normalizing to loading control (α-tubulin) and the control for each experiment. Data were collected from three independent experiments.

### RT–PCR and real-time PCR

Total RNAs were extracted from 22–32 mg of hippocampi of homozygous and heterozygous GSG1L KO by using RNeasy Mini Kit (QIAGEN 74104) according to manufacturer's instructions. RNase inhibitor (Invitrogen N8080119) was supplemented, and cDNAs were obtained by using SuperScript II Reverse Transcriptase (Invitrogen 18064-014) and Random hexamers (Invitrogen N808–0127). PCR primers were designed to amplify a 156 bp fragment across exon 3 and 4 of predicted rat GSG1L mRNA (XM_574558.3). GSG1L Forward primer: 5′- CGTCCGTCACTACGCTCAAC -3′; GSG1L Reverse primer: 5′- CACTCACGTCCCCCTTCTC -3′. A 245 bp fragment of beta-actin (Forward primer: 5′- GTGACGTTGACATCCGTAAAGA -3′); (Reverse primer: 5′- GCCGGACTCATCGTACTCC -3′) was included as a loading control. QuantiTect SYBR Green PCR Kit (QIAGEN 204143) was used in quantitative real-time reverse transcription–PCR (qRT–PCR) experiments by following manufacturer's instruction. The signals were detected by 7900HT real-time cycler (Applied Biosystems) and relative expression levels were calculated by using ΔΔ*C*_T_ method. For statistical analysis, an unpaired two-tailed *t*-test was used. The data were presented as mean±s.e.m., and were considered significant when the *P* value<0.05, 0.01 or 0.001 (indicated as *, ** or ***, respectively).

### Immunocytochemistry in neurons

Rat hippocampal dissociated neuronal cultures were performed as described^64^. Neuronal cultures were transfected at DIV14–15 using 1.5 μg of plasmid DNA with 0.75 μl Lipofectamine 2000 (Life Technologies) per well. Endogenously expressed sGluA1 in live neurons (DIV 17–18, 3 days post-transfection) was labelled using mouse monoclonal antibody against an extracellular epitope of GluA1 (clone RH95; 5 μg ml^−1^) for 10 min at 37 °C in culture media. Neurons were washed in artificial cerebrospinal fluid (ACSF; 10 mM HEPES, 150 mM NaCl, 3 mM KCl, 10 mM glucose, 2 mM CaCl_2_, 1 mM MgCl_2_, pH adjusted with CsOH to 7.35), fixed with ice-cold 4% paraformaldehyde (PFA)/4% sucrose for 15 min. Without permeabilization, cells were blocked for 30 min in 1 × PBS containing 10% normal goat serum (NGS) (Vector Laboratories, Burlingame, CA, USA), followed by Alexa647-conjugated secondary antibody (Molecular Probe). After permeabilization using 0.1% Triton X-100 in 1 × PBS for 15 min at room temperature, and blocking for 30 min in 1 × PBS containing 10% NGS and 10 μg ml^−1^ unconjugated goat anti-mouse IgG (to block any remaining GluA1-coupled antibody), Myc-tagged GSG1L was labelled using mouse monoclonal anti-c-Myc (Cell Signaling Technology 2276; clone 9B11; 1:500) in 1 × PBS/3% NGS for 30 min at room temperature before labelling with Alexa Fluor 555-conjugated goat anti-mouse secondary antibody (Molecular Probe). For sGluA1/GluA2 and total GluA1/GluA2 experiments in GSG1L KO rat, total GluA1 was labelled after permeabilization with rabbit polyclonal antibody against C-terminal of GluA1 (Millipore AB1504, 1:1,000). Coverslips were mounted with Fluoromount G (Southern Biotech).

### Internalization and recycling assay

GSG1L-Myc or GSG1L–GFP plasmids were transfected into cultured hippocampal neurons prepared from wild-type rat. Three days after transfection, neurons were incubated live with anti-GluA1 or anti-GluA2 N-terminal antibody (Millipore, MAB397) for 10 min in culture medium. For internalization assays, neurons were washed with fresh medium and then incubated in cultured medium for 10–30 min to allow internalization. After wash and fixation, sGluA1 were stained with anti-mouse Alexa Fluor 555-conjugated goat anti-mouse secondary antibody. Internalized GluA1 was labelled with Alexa Fluor 488-conjugated goat anti-mouse secondary antibody or Alexa Fluor 633-conjugated goat anti-mouse secondary antibody (for GSG1L–GFP transfected neuron only) after permeabilization in PBS containing 0.3% Triton X-100. For recycling assays, antibodies were washed off with warm ACSF after incubation at 37 °C for 10 min to allow internalization. Neurons were incubated with goat anti-mouse secondary antibody (horseradish peroxidase-conjugated, non-fluorescence) for 20 min at room temperature to block remaining sGluA1. Then neurons were washed and incubated in culture medium at 37 °C for 30 min to allow recycling. Recycled sGluA1 and internalized GluA1 were labelled the same way as described in internalization assay. Images were analysed by imageJ and data were presented as a ratio of intracellular/surface intensities (mean±s.e.m.).

### Image acquisition

For sGluA1 intensity image acquisition in Fig. 2e, images were acquired on a Zeiss LSM 710 laser scanning confocal microscope using a × 63 oil objective (1.4 numerical aperture) following the procedure described before (ref. 65). Image acquisition was performed using identical settings for a particular experiment. Images were captured using a resolution of 1,024 × 1,024 and a digital zoom of 1.0, a pixel dwell time of 0.79 μs, each lines were averaged four times. Multiple confocal slices were collected with step intervals of 0.37 μm in the *z* direction to image potential out of focus dendrites. For surface/total GluA1 staining in dissociated hippocampal neuronal culture, images were acquired on a Zeiss LSM 510 laser scanning confocal microscope using a × 63 oil objective (1.4 numerical aperture). Multiple *z* sections (nine optical slices) of dendrites were acquired at 0.5 μm. Images were captured using a 1,024 × 1,024 pixel screen and gains for both fluorophores were between 700 and 800. Scan speed function were set to 9 and the mean of four lines was collected. For the fluorescence intensity of GSG1L–GFP or its mutants in cultured organotypic slices, images were acquired on the Zeiss LSM 510 confocal microscope using a × 20 air Planneofluar objective. Multiple *z* sections of neuronal soma or secondary apical dendrites were collected at 1.0-μm with 512 × 512 pixel screen. Pinhole was set to 1 airy unit for all experiments. Laser power, digital gain and offset settings were all identical in each experiment by using the ‘reuse' function in LSM software.

### Images analysis

For quantitative analysis of fluorescent immunostained sGluA1 (Fig. 2e), maximal projection images were created with the ZEN software (Zeiss) from 4–6 serial optical sections. Using Metamorph (Universal Imaging Corp., Downingtown, PA, USA), quantitation of the fluorescence signal from sGluA1 was determined from fluorescent signal above a threshold set for distinguishing dendritic morphology from background. The threshold value was held identical within single experiment, and only slightly adjusted between independent experiments. For each image collected containing one GSG1L-transfected neuron and non-transfected neurons, dendritic outline was drawn to cover 25–30 μm in length (representing a surface area of 850–1,000 pixels). The integrated fluorescent intensity of sGluA1 was calculated from one segment of dendrite positive for Myc-tagged GSG1L and from one GSG1L-negative neighbouring dendrite section. We performed 6 experiments from 6 independent hippocampal neurons preparation, and 5–15 images were analysed for each independent experiment. For surface/total ratios of GluA1 staining in dissociated hippocampal neuronal cultures, and GFP fluorescent signals in organotypic slice cultures, maximal projection images were generated by the LSM 510 Browser software. Background was subtracted by using ‘subtract background' function in ImageJ software, and the background level was held identical for all cells within each experiment. Region-of-interest (ROI) was defined along a segment of the dendrite 25–30 μm, or neuronal soma according to the fluorescence signal distinguished from the background in ImageJ software. Average values of fluorescence intensities in ROI (the total fluorescent intensity divided by the total area of a dendritic segment) were calculated by ImageJ. For GluA1 puncta analysis ROI was defined along a segment of the dendrite 25–30 μm. Images were set to a threshold that can distinguish the GluA1 puncta from the background. Both the length and the area of the selected ROI of the dendrites were measured. The number of GluA1 puncta (3–83 pixels in size) in the ROI was determined by the ‘Analyse Particles' function in ImageJ software. The density of the GluA1 puncta was determined by dividing the total number of puncta by the area of the selected ROI. For GFP fluorescence signals in the WT GSG1L, the GSG1l mutants, the Claudin1 mutant and the γ8 mutants, 5–10 neurons were analysed.

Statistical analysis was performed using GraphPad Prism 6, and statistical significance between conditions was calculated using the two-tailed, unpaired *t*-test or One-way analysis of variance (ANOVA) test as indicated in figure legends. Significance was considered when the *P* value was <0.05, 0.01 or 0.001 (indicated as *, ** or ***, respectively). The data were presented as mean±s.e.m.

### Neuronal spine analysis

For spine analysis, GSG1L was expressed for the same amount of time as for those electrophysiological recordings and they were from the same batches of hippocampal slice cultures and underwent the same procedure of gene-gun mediated transfection. CA1 pyramidal cells in hippocampal organotypic slice cultures were filled with Alexa Fluor 568 dyes through the patch pipette for about 10 min. After filling, slices were fixed in 4% PFA/4% sucrose in PBS for 30 min at room temperature, followed by washing three times with 1 × PBS. Slices were mounted and imaged by using a Zeiss LSM 510 confocal laser scanning microscope. For spine analysis, three-dimensional (3D) stacks of a 20-μm dendritic stretch of each neuron from secondary apical dendrites were collected by using a × 63 oil immersion lens, and spines were counted in 3D projection mode by using Zeiss software. For statistical analysis, an unpaired two-tailed *t*-test was used. The data were presented as mean±s.e.m., and were considered significant when the *P* value<0.05.

### Electrophysiology in neurons

Transverse 300 μm hippocampal slices were cut from P13–P19 WT and GSG1L KO rats on a DSK linear slicer Pro7 vibratome in cutting solution containing (in mM) KCl 2.5, CaCl_2_ 0.5, MgCl_2_ 7, NaH_2_PO_4_ 1.25, NaHCO_3_ 25, glucose 7, ascorbic acid 1.3 and sucrose 210. Freshly cut slices were placed in an incubating chamber containing ACSF, containing (in mM) NaCl 119, KCl 2.5, NaHCO_3_ 26.2, NaH_2_PO_4_ 1, glucose 11, CaCl_2_ 2.5 and MgSO_4_ 1.3, and recovered at 32 °C for 30–60 min. Slices were then maintained in ACSF at room temperature prior to recording. After 30–60 min of incubation at room temperature, slices were transferred to a submersion chamber on an upright Olympus microscope, perfused in normal ACSF with picrotoxin (100 μM), and saturated with 95% O_2_/5% CO_2_. The intracellular solution contained (in mM) CsMeSO_4_ 135, NaCl 8, HEPEs 10, Na_3_GTP 0.3, MgATP 4, EGTA 0.3, QX-314 5 and spermine 0.1. Cells were recorded with 3- to 5-MΩ borosilicate glass pipettes. Series resistance was monitored and not compensated, and cells in which series resistance varied by 25% during a recording session were discarded. Synaptic responses were collected with a Multiclamp 700B amplifier (Axon Instruments, Foster City, CA), filtered at 2 kHz and digitized at 10 kHz. All pharmacological reagents were purchased from Abcam, and other chemicals were purchased from Sigma.

For LTP recording at Schaffer-collateral/CA1 synapses in acute hippocampal slices prepared from WT and KO rats, recording pipette was filled with ACSF and placed in stratum radiatum of the CA1 region, and stimulation of Schaffer collaterals was performed with monopolar glass electrodes filled with ACSF and placed in stratum radiatum at the CA1 region at a distance of ∼250 μm away from the recording electrode. For whole-cell LTP recording, EPSCs were recorded at −70 mV for 3–5 min as baseline before LTP induction. Whole-cell LTP was induced within 5 min after rupture of the patch membrane by pairing stimulation at 2 Hz for 1 min with depolarization to 0 mV. Evoked AMPA EPSCs in acute hippocampal slices were recording at −70 mV and evoked NMDA EPSCs were recording at +40 mV and measured at 100 ms after stimulation where AMPA EPSCs have completely decayed. Mean EPSCs were an average of 20–50 sweeps. mEPSCs were measured at −70 mV in the presence of 0.5–1 μM TTX.

Organotypic hippocampal slice cultures were prepared and transfected as previously described^34^. Briefly, hippocampi were dissected from P6–P8 WT mice, *Gria1–3*^*f/f*^ mice or P6–P8 *Gsg1l* mutant rats, and transfected biolistically with plasmids 3–4 days after in culture. Slices were cultured for additional 2–5 days (protein overexpression) or 7–12 days (protein knockdown) before recording for cultures prepared from WT mice and *Gsg1l* rat KOs, or 14–18 days before recording for cultures prepared from *Gria1–3*^*f/f*^ mice. For all recording in slice cultures, ACSF was modified to contain 4 mM CaCl_2_ and 4 mM MgSO_4_. For recording evoked EPSCs in organotypic slices, ACSF was also supplemented with 5–20 μM 2-chloroadenosine to dampen epileptiform activity, and GABA_A_ receptors were blocked by picrotoxin (100 μM). Synaptic responses were similarly recorded as described above in acute slices. Mice housing, breeding and handling protocols were approved by NINDS ACUC at NIH. Mice of both sexes at the age of p6–p8 were used for organotypic hippocampal slice cultures.

GFP-positive neurons in organotypic slice cultures were identified by epifluorescence microscopy. All paired recordings involved simultaneous whole-cell recordings from one GFP-positive neuron and a neighbouring GFP-negative control neuron. The stimulus was adjusted to evoke a measurable, monosynaptic EPSC in both cells. AMPA EPSCs were measured at a holding potential of −70 mV, and NMDA EPSCs were measured at +40 mV and at 100 ms after the stimulus, at which point the AMPA EPSC has completely decayed. In the scatter plots for simultaneous dual recordings, each open circle represents one paired recording, and the closed circle represents the average of all paired recordings. In the scatter plot, the *x*-axis represents the EPSC recorded in the control cell, and the *y*-axis represents the EPSC recorded in the transfected cell. Virtual 1:1 diagonal line is also shown. If the data point falls above the diagonal line, it indicates that the EPSC is higher in the transfected cell. If it falls below the diagonal line, it indicates that the EPSC is higher in the control cell. AMPAR-mediated currents from somatic outside-out patches were recorded at −70 mV by local application of 1 mM glutamate and 100 μM cyclothiazide, in presence of 100 μM D-APV, 0.5 μm TTX and 100 μM picrotoxin, for 2 s. Rectification index values were calculated as the ratio of the slopes of the two lines connecting current amplitudes at −70, 0, and +40 mV. Paired-pulse ratios were measured by giving two pulses at a 50-ms interval and taking the ratio of the two peaks of the EPSCs from an average of 20–50 sweeps. mEPSCs were acquired in the presence of 0.5–1 μM TTX and sEPSCs were acquired in the absence of TTX, and were semiautomatically detected by offline analysis using in-house software in Igor Pro (Wavemetrics) developed in Dr Roger Nicoll's laboratory at UCSF, using an amplitude threshold of 6 pA. All events were visually inspected to ensure they were m/sEPSCs during analysis and those non-m/sEPSC traces were discarded (the recording noise was ∼6 pA). All paired recording data were analysed statistically with a two-tailed paired Student *t*-test. For all other analyses, an unpaired two-tailed *t*-test was used. Kolmogorov–Smirnov test was used for m/sEPSC cumulative distributions. The data were presented as mean±s.e.m., and statistical significance was defined as *P*<0.05, 0.01 or 0.001 (indicated as *, ** or ***, respectively). *P* values ⩾0.05 were considered not significant.

### HEK cell culture and transfection for electrophysiological analysis

HEK (HEK293T) cells were used for expression of GluA1, GSG1L, GluA1/GSG1L and CNIH2. Transfection was performed in 24-well plates with indicated cDNAs using Effectene transfection reagents according to the protocol provided by the manufacturer, and suspension transfection strategies were used. Total cDNA (0.4 μg, including 0.2 μg of GluA1, the corresponding amount of other plasmids based on the transfection ratio and the empty pcDNA3.0 plasmid used to make a total amount of 0.4 μg) used for transfection was divided into 6 wells of a 24-well plate. When co-expression was carried out, the 8:1 and 4:1 ratios of GluA1 to GSG1L and GluA1 to CNIH2, respectively, were used (the amount of GluA1 was always 0.2 μg). And an 8:2:1 ratio of GluA1, CNIH2 and GSG1L was used when three constructs were co-expressed (the amount of GluA1 was 0.2 μg). For GluA1/GSG1L fusion plasmid and CNIH2 co-expression, a 4:1 (GluA1/GSG1L:CNIH2) ratio was used for transfection (the amount of GluA1/GSG1L was 0.2 μg). We chose these ratios after a few pilot experiments indicated that these ratios of transfections allowed us to record sizable AMPAR currents and at the same time also kept the cells healthy. Before transfection, HEK cells were dissociated with 0.25% trypsin-EDTA and plated on 12-mm coverslips pretreated with poly-D-lysine. Total cDNA was added to cell suspension and waited for 3–4 h, then replaced with fresh medium containing 100 μM NBQX. Recording was performed 48–72 h after transfection.

### AMPAR kinetic analysis in HEK cells and in neurons

Outside-out patches were excised from transfected HEK cells or hippocampal neurons in acute slices or slice cultures. Coverslips with transfected HEK cells were maintained during recording with external solution containing (in mM) NaCl 140, KCl 5, MgCl_2_ 1.4, EGTA 5, HEPES 10, NaH_2_PO_4_ 1, D-glucose 10 and NBQX 0.01, with pH adjusted to 7.4. Outside-out patches were excised from positively transfected cells identified by fluorescence microscopy with 4–6 MΩ borosilicate glass pipettes. The internal solution contained (in mM) CsF 135, CsOH 33, MgCl_2_ 2, CaCl_2_ 1, EGTA 11, HEPES 10 and spermine 0.1, with pH adjusted to 7.2. Glutamate (1 mM) was dissolved in external solution, and the glutamate-evoked currents were recorded while holding the patches at −70 mV. The hippocampal slices (acute or culture) were perfused with ACSF bubbled with 5% CO_2_ and 95% O_2_. In slice cultures, the cells with GSG1L overexpression were visualized with EGFP fluorescence. Outside-out patches were excised from CA1 pyramidal neurons and hippocampal dentate granule neurons. The currents were recorded with pipette solution containing (in mM) CsMeSO_4_ 135, NaCl 8, HEPES 10, Na_3_GTP 0.3, MgATP 4, EGTA 0.3, QX-314 5 and spermine 0.1. Glutamate was similarly dissolved in HEK extracellular solution with the addition of 50 μM D-APV, 0.5 μM TTX and 100 μM picrotoxin to isolate AMPAR-mediated currents. Rapid application/removal of glutamate (every 5 s) was performed using a Piezo-controlled fast application system with a double-barrel application pipette that enables solution exchange. The glutamate-evoked currents were recorded while holding the patches at −70 mV. Data were collected with a Multiclamp 700B amplifier, filtered at 2 kHz and digitized at 50 kHz.

Typically, outside-out patch currents were averaged from 6 to 10 traces. We noticed that some traces were well-fit to a monoexponential equation while some other traces were best fit to a double-exponential function. To simplify the comparison of decay time constants across different experimental conditions (the kinetics of deactivation, desensitization, evoked EPSCs and mEPSCs of AMPARs), a single weighted decay measure was calculated from the area under the peak-normalized current^66^, according to 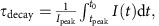 where *t*_0_ was 60 ms after the peak^23^. The recovery from desensitization of AMPARs were carried out with paired-pulse protocol consists of a 100-ms pulse followed by a 10-ms pulse in an increasing interval. The peak currents recorded during the second pulse divided by the maximal current recorded during the first pulse was considered as the recovery rate at the different time points. The recovery from desensitization was characterized by time constants derived from a monoexponential fit. Curve fitting and data analysis were done with Igor Pro 6.22A. It is worth noting that our overexpression experiments were performed in mouse slice cultures and gene KO experiments were performed in acute rat hippocampal slices. Thus, the *τ* values for deactivation and desensitization in rat or mouse hippocampal CA1 neurons are different, but are consistent with published data^10^,^22^,^24^,^59^,^67^,^68^. It is also worth noting that AMPAR deactivation kinetics was slightly faster in older hippocampal slice cultures than that in younger hippocampal slice cultures (Figs 6a,7e), although the difference was not significant (two-tailed *t*-test, *P*=0.3 between control *τ* numbers, *P*=0.22 between GSG1L *τ* numbers). Such developmental speeding of AMPAR deactivation kinetics has been observed in many CNS neurons^66^,^69^,^70^. We performed these experiments in Fig. 7e in older cultures because we had to wait for enough time for shRNA knockdown of CNIH2 (over a week), and thus GSG1L was also overexpressed for a longer time in this experiment. For all other overexpression experiments without shRNA knockdown, the constructs were expressed for 2–5 days before electrophysiological recordings. Differences in means were tested with unpaired two-tailed *t*-test or one-way ANOVA as indicated in figure legends and statistical significance was defined as *P*<0.05, 0.01 or 0.001 (indicated as *, ** or ***, respectively). *P* values⩾0.05 were considered not significant. The data were presented as mean±s.e.m.

### Glutamate/Kainic acid puffing experiments

For glutamate-induced whole-cell currents in HEK cells, cells were cultured and transfected with GluA1, GluA1 plus GSG1L (ratio at 1:1) and GluA1/GSG1L (the cDNA amount for GluA1 or GluA1/GSG1L is the same, 0.2 μg, and an empty plasmid was used to make the total cDNA amount of 0.4 μg per transfection). Glutamate-induced whole-cell currents in HEK cells were recorded at −70 mV by local application of 1 mM glutamate and 100 μM cyclothiazide, in presence of 10 μM NBQX in the external solution. The tip of Mini-manfold was placed at ∼100 μM away from the recorded HEK cells. For the whole-cell currents in neurons evoked by Kainate or glutamate, dual whole recording were performed. The tip of the Mini-manfold was placed at ∼100 μM away from the recorded neurons. Kainate (1 mM, Abcam) and glutamate (1 mM, Sigma) solutions containing 100 μM cyclothiazide (Abcam), 0.5 μM TTX, 100 μM D-APV and 100 μM picrotoxin were sequentially applied for 0.5 s with an interval of 50 s. ACSF also contained 100 μM D-APV, 0.5 μm TTX, 100 μM picrotoxin and 10 μM NBQX (to facilitate the decay of the AMPAR-mediated whole-cell currents). *I*_Ka_/*I*_Glu_ ratios were calculated by dividing the peak current evoked by Kainate with peak current evoked by Glutamate. The data were presented as mean±s.e.m.

### Behavioural analysis

All rats were bred and housed in a conventional vivarium at the National Institutes of Health, Bethesda, Maryland, USA. Pups were kept with the dam until weaning at postnatal day 21. After weaning, juveniles were group housed by sex in standard plastic cages in groups not exceeding four per cage. Cages were maintained in ventilated racks in a temperature (20 °C) and humidity (∼55%) controlled vivarium on a 12-h circadian cycle, lights on from 0600 to 1800 hours. Standard rodent chow and reverse-osmosis water were available ad libitum. In addition to standard bedding, a cardboard tube was provided in each cage. All behavioural testing was performed with male rats aged 3–5 months. All procedures were approved by the National Institute of Mental Health Animal Care and Use Committee.

### Morris water maze

Hippocampus-dependent spatial memory was assessed through the Morris water maze task, a widely used measure of spatial memory. A circular pool (120 cm diameter) made of white plastic was filled to a depth of ∼45 cm with room-temperature water made opaque by the addition of non-toxic white tempera paint. A hidden escape platform (clear plastic; 12 × 12 cm square) was submerged ∼3 cm below the surface of the water. The pool was divided into four quadrants, and the platform was placed in the centre of one quadrant, with the location of this target quadrant counterbalanced across groups. Animals had no swim experience prior to the start of training. Each animal received four trials per day for 5 consecutive days, with an intertrial-interval (ITI) of 2 min between trials. Rats were placed into the pool facing the side wall and allowed to swim until they found the platform, or for a maximum of 90 s. Semi-random start positions were used, such that the four start positions were designated in the quadrants of the pool that did not contain the platform, with the restriction that one trial each day is from each of the four positions. Any rat that failed to find the platform within this time was guided to the platform by the experimenter. After reaching the platform, the animal was allowed to remain there for 30 s before being removed from the pool. The animal then remained on the platform for 30 s before commencing the next trial. On the 6th day, 24 h after the final acquisition trial, a probe trial was conducted to assess spatial reference memory. In this trial, the platform was removed from the pool and the rats were given 90 s to swim. The percentage of time that animals spent in the quadrant of the pool previously containing the platform was used as a measure of spatial memory retention. Automated video tracking software (AnyMaze, Stoelting Co.) was used to record latency to reach the platform zone, distance travelled, swim speed, path efficiency (actual path length/direct path length) and time spent in each of the four quadrants of the pool on all trials. During the 5 days of acquisition training, data from each of the four training sessions were averaged for each animal to give average daily performance scores. Data analysis was performed using the statistical software SPSS 18.0 for Windows. The five acquisition training days were analysed with a two-way ANOVA with Strain (WT and KO) as the between-subjects factor, and the within-subjects factor of Day (days 1–5 in the acquisition phase). For the probe trial, time spent in each quadrant was analysed using a two-way ANOVA with the between-subjects factor of Strain (WT and KO) and the within-subjects factor of Quadrant (quadrants a, b, c and d), and unpaired *t*-tests were used to compare time spent in the target quadrant for the two strains. For all tests, results were considered significant at *P*<0.05.

### Object recognition

The object recognition tasks were used to assess an animal's ability to recognize an object with either in a novel spatial location or with a novel physical appearance. This test utilizes the natural inclination for a rodent to spend more time interacting with an object that is novel in either its appearance or location over a familiar one. Both object recognition tests were performed in a square white plastic box (58 × 58 × 38 cm) under low light levels (∼120 lux). Animals were handled daily for 1 week and then habituated to the empty test apparatus for 15 min per day for 3 consecutive days prior to the start of recognition testing. These habituation sessions were used to acclimate the animals to the testing procedures as well as to assess baseline motor activity. Object recognition testing took place in two stages: novel object recognition and spatial object recognition. Both assays had two phases, the familiarization phase and the probe test. In the novel object recognition test, during the familiarization phase two identical objects (A1 and A2) were placed in the apparatus 15 cm away from two adjacent corners of the box. Objects were of sufficient weight and size to ensure they could not be moved or knocked over by the animals. The rats were placed in the chamber facing the wall farthest from the objects and allowed to explore the apparatus and objects for 10 min. Following the familiarization phase, animals were returned to their home cage for a 1-h ITI. Subjects were then reintroduced to the apparatus for 5 min for the probe phase. During the test phase, the apparatus contained one object identical to those used in the familiarization trial (A3) and one novel object (B1) that were placed in the same spatial location as the objects used in the habituation phase. All objects used were approximately the same size and shape, but differed in colour and textural characteristics. The identity and position of the novel and familiar objects were counterbalanced across groups. Following each trial, the arena and objects were cleaned with 70% ethanol to minimize olfactory cues. The spatial object recognition test was performed 2 weeks after the completion of the novel object recognition assessment. The same subjects and apparatus were used, and the objects were assigned so that each subject was exposed to different objects in the novel object and spatial recognition tests. In the spatial recognition assessment, during the 15-min familiarization phase two identical objects (A1 and A2) were placed in the apparatus. After a 1-h ITI, the animal was returned to the apparatus for the 5-min probe test. In this test, the same two objects from the familiarization test were presented to the animal, with one object in the same spatial location (*A*_Stationary_) and the other moved to a novel spatial location (*A*_Moved_). The potential object locations for both phases were 15 cm away from each corner of the box and were randomized across subjects. Video tracking software was used to record exploratory behaviours. Exploration time was defined as the animal's nose being within 2 cm of the object and included behaviours such as licking, sniffing or touching the object. For both the novel object and spatial recognition tests, object exploration time during the familiarization and test phases was analysed with a mixed-model ANOVA with the between-subjects factor of Strain (WT or KO) and the within-subjects factors of Test (Familiarization or Probe) and Object (A1, A2, A3 or B1 for novel object recognition; A1, A2, *A*_Stationary_, or *A*_Moved_ for the spatial object recognition assessment). Additional analysis on main effects and interactions were performed with Tukey's *post hoc* tests or pair-wise analysis with Bonferroni corrections for multiple comparisons. Significance level for all tests was set at *P*<0.05. The data were presented as mean±s.e.m.

## Additional information

**How to cite this article:** Gu, X. *et al*. GSG1L suppresses AMPA receptor-mediated synaptic transmission and uniquely modulates AMPA receptor kinetics in hippocampal neurons. *Nat. Commun.* 7:10873 doi: 10.1038/ncomms10873 (2016).

## Supplementary Material

Supplementary Information Supplementary Figures 1-15
