# Supplementary material for: GSG1L suppresses AMPA receptor-mediated synaptic transmission and uniquely modulates AMPA receptor kinetics in hippocampal neurons
Source: Nat Commun. 2016 Mar 2;7:10873. doi: 10.1038/ncomms10873 (PMC4778064; doi:10.1038/ncomms10873)
Supplement: Supplementary Information — Supplementary Figures 1-15 [file ncomms10873-s1.pdf]

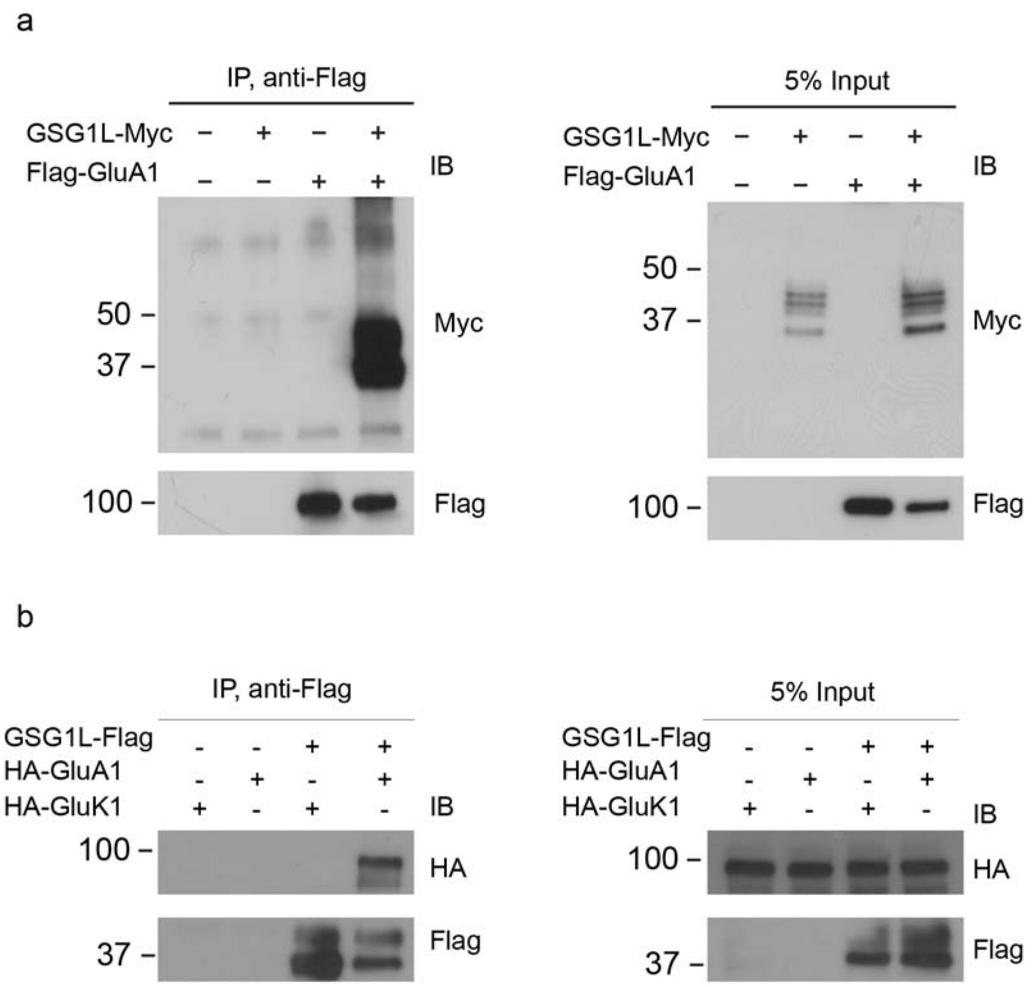

Supplementary Fig.1. Co-immunoprecipitation of GSG1L with GluA1 or GluK1 in HEK cells.

(a) GSG1L-Myc and Flag-GluA1 were expressed alone or together in HEK cells. Flag-tagged GluA1 in cell lysates was immunoprecipitated with an anti-Flag antibody and immunoprecipitates (IP) were subjected to a western blotting assay with anti-Myc and anti-Flag antibodies, respectively (left). 5% of the lysate used for immunoprecipitation (input) was also probed with indicated antibodies to confirm protein expression (right). (b) GSG1L-Flag and HA-GluA1 or HA-GluK1 were expressed in HEK cells. GSG1L were immunpreciated with anti-Flag antibody, and GluA1 and GluK1 were probed with anti-HA antibody.

#

#

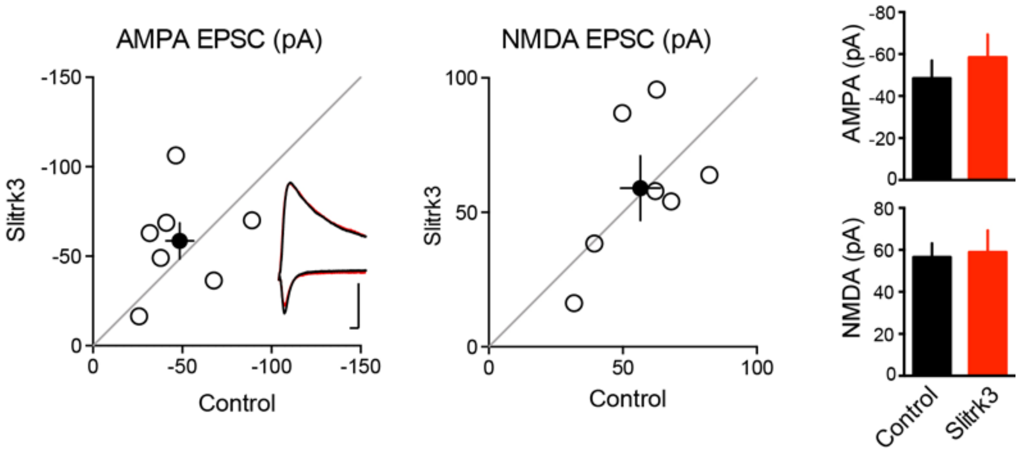

Supplementary Fig.2. Overexpression of Slitrk3 in hippocampal CA1 pyramidal neurons does not alter AMPA or NMDA EPSCs

Scatter plot of AMPAR and NMDAR-mediated EPSCs in neurons expressing Slitrk3 (Slitrk3-IRES-mCherry) and nearby control neurons. Overexpression of Slitrk3 in mouse organotypic hippocampal slice cultures did not affect either AMPA or NMDA EPSCs in CA1 pyramidal neurons (AMPA: n = 7; p = 0.58; NMDA: n = 7; p = 0.94; paired t-test). Scale bar: 20 pA and 20 ms. All data were presented as mean ± SEM.

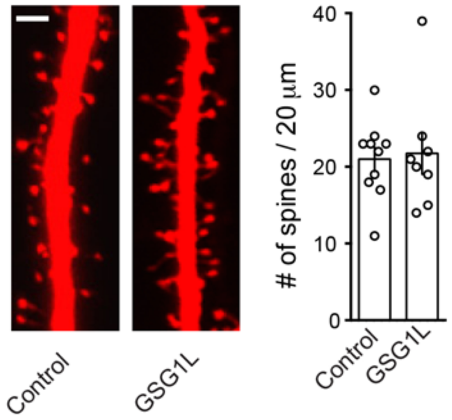

Supplementary Fig.3. Overexpression of GSG1L does not affect the number of dendritic spines

There was no change of spine density in CA1 pyramidal neurons overexpressing GSG1L in cultured organotypic hippocampal slices (control, n = 10; GSG1L,; n = 8; p = 0.81; *t*-test). All data were presented as mean ± SEM, Scale bar: 5 µm.

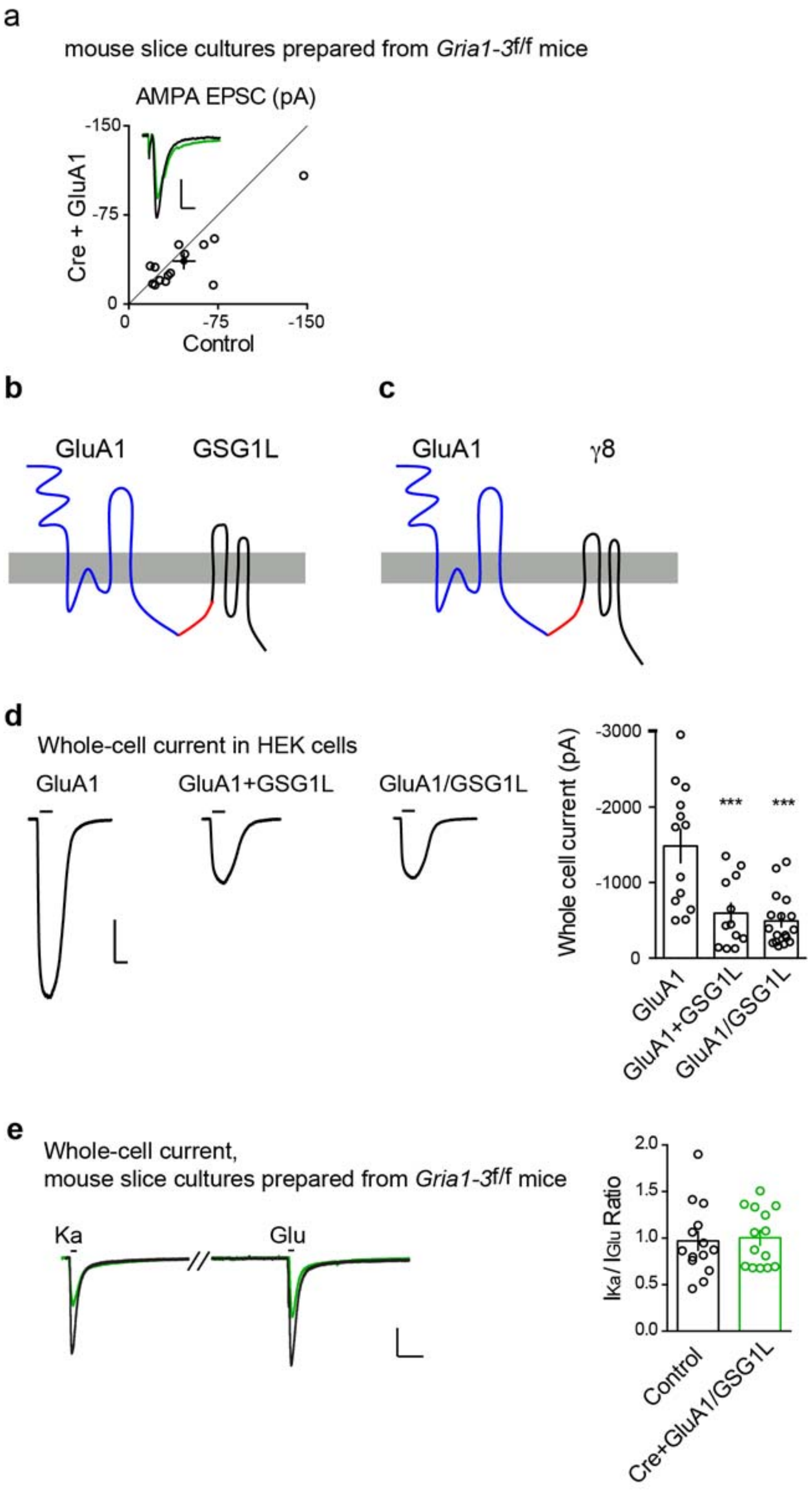
a
mouse slice cultures prepared from Gria1-3f/f mice
AMPA EPSC (pA)
-150
-75
0
Cre + GluA1
0
-75
-150
Control
b
GluA1
GSG1L
c
GluA1
γ8
d
Whole-cell current in HEK cells
GluA1
GluA1+GSG1L
GluA1/GSG1L
-3000
-2000
-1000
0
Whole cell current (pA)
***
***
GluA1
GluA1+GSG1L
GluA1/GSG1L
e
Whole-cell current,
mouse slice cultures prepared from Gria1-3f/f mice
Ka
Glu
2.0
1.5
1.0
0.5
0.0
IKa/ IGlu Ratio
Control
Cre+GluA1/GSG1L

Supplementary Fig. 4. Characterization of GluA1/GSG1L fusion plasmid

(a) GluA1 rescued ~75% AMPA EPSCs in CA1 pyramidal neurons expressing Cre in hippocampal slice cultures prepared from *Gria1-3*$^{f/f}$ mice (AMPA: n = 14 pairs ; p = 0.06). Scale bar: 20 pA and 20 ms.

(**b-c**) Schematic drawings of GluA1 fusion to GSG1L (**b**, GluA1/GSG1L) and GluA1 fusion to γ8 (**c**) GluA1 is shown in blue, GSG1L or γ8 is shown in black and the linker sequence is shown in red.

(**d**) Glutamate-evoked AMPAR-mediated whole-cell currents (1 mM glutamate in the presence of 100 μM cyclothiazide) in HEK cells expressing GluA1, GluA1 plus GSG1L or GluA1/GSG1L. Co-expression of GSG1L with GluA1 or expression of GluA1/GSG1L fusion protein reduced AMPAR-mediated whole-cell currents (GluA1: n = 13; GluA1 + GSG1L: n = 12; GluA1/GSG1L: n = 17; One-way ANOVA test, p < 0.001). Scale bar: 500 pA and 0.5 s.

(**e**) There was no difference of I_{Ka}/I_{Glu} ratios between control CA1 pyramidal neurons and neurons expressing Cre and GluA1/GSG1L in hippocampal slice cultures prepared from *Gria1-3*$^{f/f}$ mice (n = 14 pairs, p = 0.8, *t*-test). Scale bar: 500 pA and 5 s.

All data were presented as mean ± SEM**.**

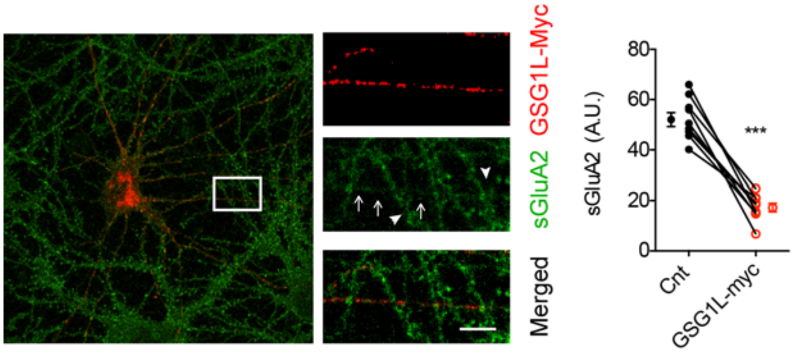

Supplementary Fig. 5. Over-expression of GSG1L-Myc reduced surface GluA2 expression in cultured hippocampal neurons.

(left) Representative image of cultured hippocampal neurons expressing Myc-tagged GSG1L (red), and stained for surface GluA2 (green). The boxed area was shown in the middle. (Middle) Arrow heads indicated the surface expression of GluA2 (sGluA2) on a non-transfected dendrite and arrows indicated the surface expression of GluA2 on a Myc-tagged GSG1L-expressing dendrite. Scale bar, 5 µm. (Right) Scatter plot represented the distribution of each pair of cells analyzed and shown that GluA2 surface expression on Myc-tagged GSG1L positive dendrites (GSG1L) was significantly reduced (n = 10 pairs of neighboring neurons; $p < 0.001$). All data were presented as mean ± SEM.

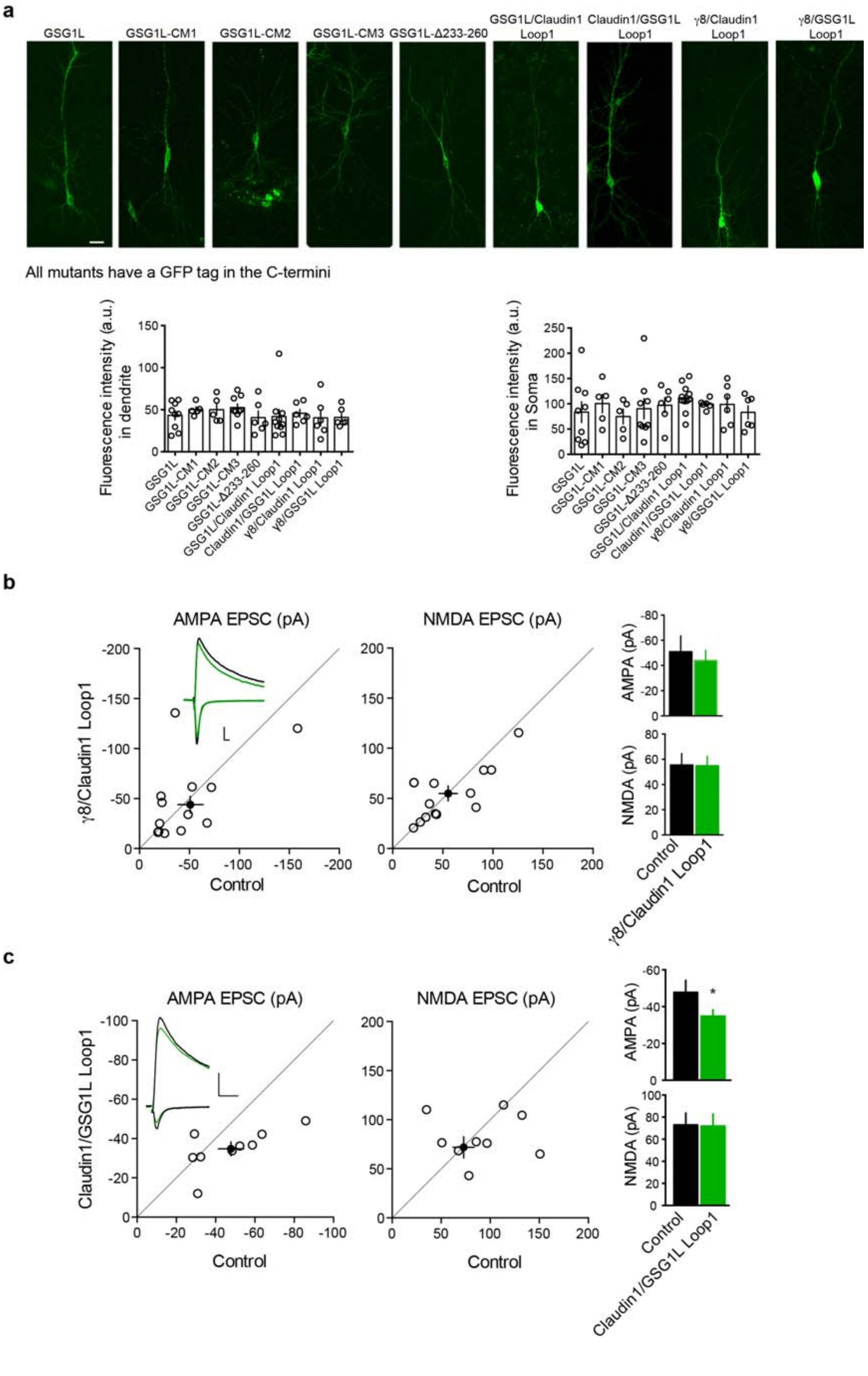
a
GSG1L
GSG1L-CM1
GSG1L-CM2
GSG1L-CM3
GSG1L-Δ233-260
GSG1L/Claudin1 Loop1
Claudin1/GSG1L Loop1
γ8/Claudin1 Loop1
γ8/GSG1L Loop1
All mutants have a GFP tag in the C-termini
Fluorescence intensity (a.u.) in dendrite
Fluorescence intensity (a.u.) in Soma
b
AMPA EPSC (pA)
NMDA EPSC (pA)
γ8/Claudin1 Loop1
Control
AMPA (pA)
NMDA (pA)
c
AMPA EPSC (pA)
NMDA EPSC (pA)
Claudin1/GSG1L Loop1
Control
AMPA (pA)
NMDA (pA)
*

#

Supplementary Fig. 6. Characterization of GSG1L mutants

(**a**) Representative images of neuron expressing GSG1L and GSG1L, Claudin1 or γ8 mutants in mouse organotypic hippocampal slice cultures (Scale bar, 20 μm). Bar graphs show no significant differences in their expression at either dendritic or somatic locations ($p > 0.05$, One-way ANOVA test).

(**b**) Over-expression of the γ8/Claudin1 Loop1 mutant did not affect either AMPA or NMDA EPSCs in cultured organotypic hippocampal slices (AMPA: $p = 0.49$; n = 13; NMDA: $p = 0.93$; n = 13; paired *t*-test). Scale bar 20 pA and 20 ms.

(**c**) Over-expression of Claudin1/GSG1L Loop 1 mutant reduced AMPA EPSCs, but not NMDA EPSCs (AMPA: $p < 0.05$; n = 9; NMDA: $p = 0.93$; n = 9; paired *t*-test). Scale bar 20 pA and 20 ms.

All data were presented as mean ± SEM**.**

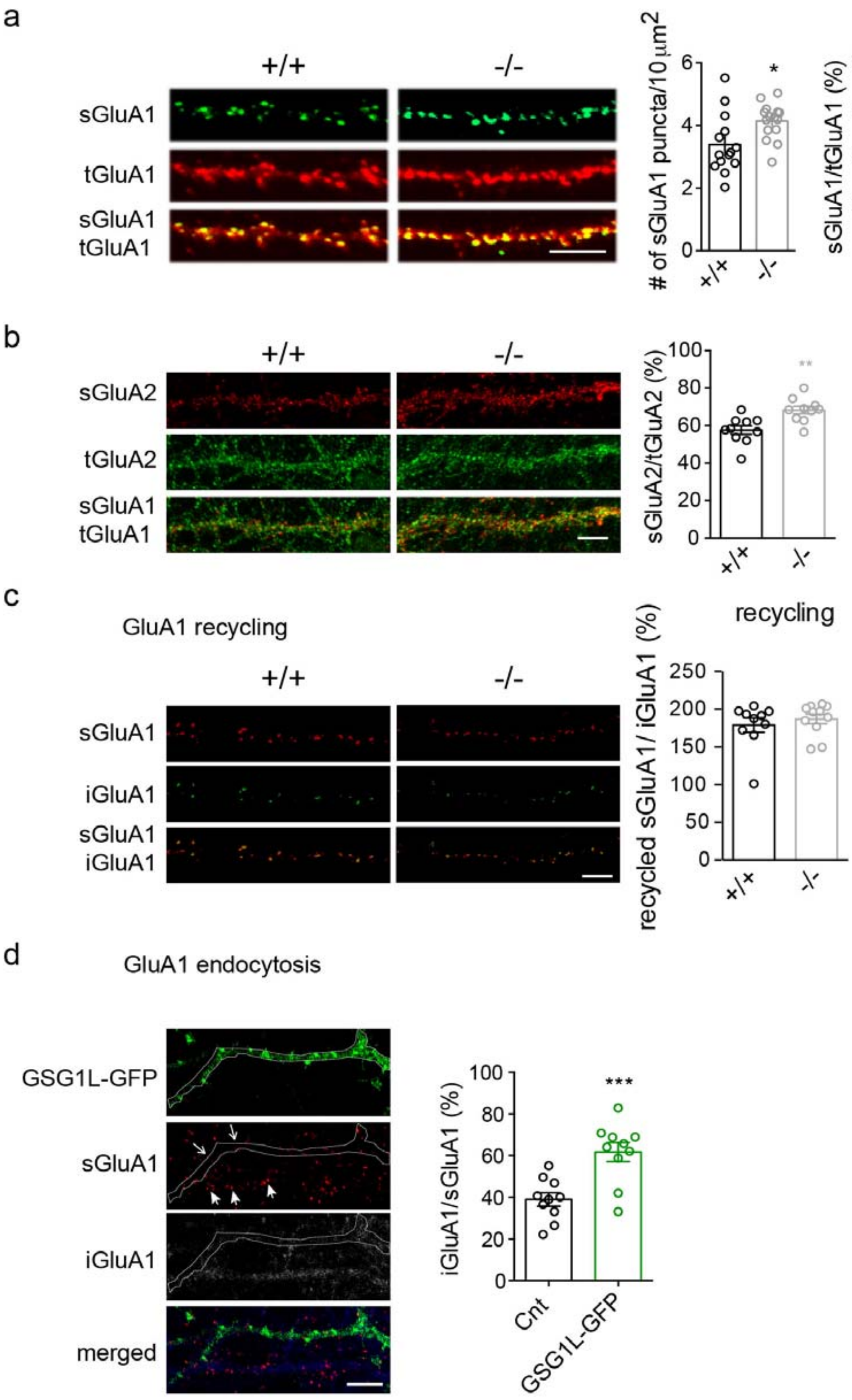
a
+/+
-/-
sGluA1
tGluA1
sGluA1
tGluA1
# of sGluA1 puncta/10 μm2
sGluA1/tGluA1 (%)
b
sGluA2
tGluA2
sGluA1
tGluA1
sGluA2/tGluA2 (%)
c
GluA1 recycling
recycling
sGluA1
iGluA1
sGluA1
iGluA1
recycled sGluA1/ iGluA1 (%)
d
GluA1 endocytosis
GSG1L-GFP
sGluA1
iGluA1
merged
iGluA1/sGluA1 (%)
Cnt
GSG1L-GFP

#

Supplementary Fig. 7. Enhanced surface GluA1 expression in GSG1L KO rat hippocampal neurons.

(a) GSG1L KO significantly increased surface GluA1 (sGluA1) expression in dissociated hippocampal neuronal cultures (average fluorescence intensity: sGluA1 (green): +/+, n = 14; -/-, n = 15; $p < 0.05$; tGluA1 (total GluA1, red): +/+, n = 14; -/-, n = 15; $p = 0.28$; sGluA1/tGluA1 ratio: +/+, n = 14; -/-, n = 15; $p < 0.05$; sGluA1 puncta / 10 μm^2: +/+, n = 14; -/-, n = 15; $p < 0.05$; *t*-test; n number stands for the number of neurons). Representative dendritic images of cultured hippocampal neurons (DIV17) prepared from WT and KO P1 rats (Left). Scale bar, 5 µm. All data were presented as mean ± SEM.

(b) Surface GluA2 expression increased significantly in cultured hippocampal neurons prepared from GSG1L KO rat (n = 10 for wild type neurons or GSG1L KO neurson; $p < 0.05$). Representative dendritic images of cultured hippocampal neurons (DIV 14) prepared from WT and KO P1 rats (Left). Scale bar, 5 µm. All data were presented as mean ± SEM.

(c) There was no difference in GluA1 recycling between WT and KO neuron (n = 10 for each; $p = 0.49$). Representative dendritic images of cultured hippocampal neurons (DIV 14) prepared from WT and KO P1 rats (Left). Scale bar, 5 µm. All data were presented as mean ± SEM.

(d) GluA1 endocytosis was significantly enhanced in GSG1L-GFP transfected neurons (n = 10 for each; $p < 0.001$). Representative dendritic images were taken from cultured hippocampal neurons (DIV14) at 10 mins after chasing. Arrow indicated GluA1 staining on dendrites from GSG1L-GFP transfected neurons and Arrow head indicated GluA1 staining on dendrites from neaby control neuron. Dash line outlined the dentrdites from GSG1L-GFP transfected neuron. Scale bar, 5 µm. All data were presented as mean ± SEM.

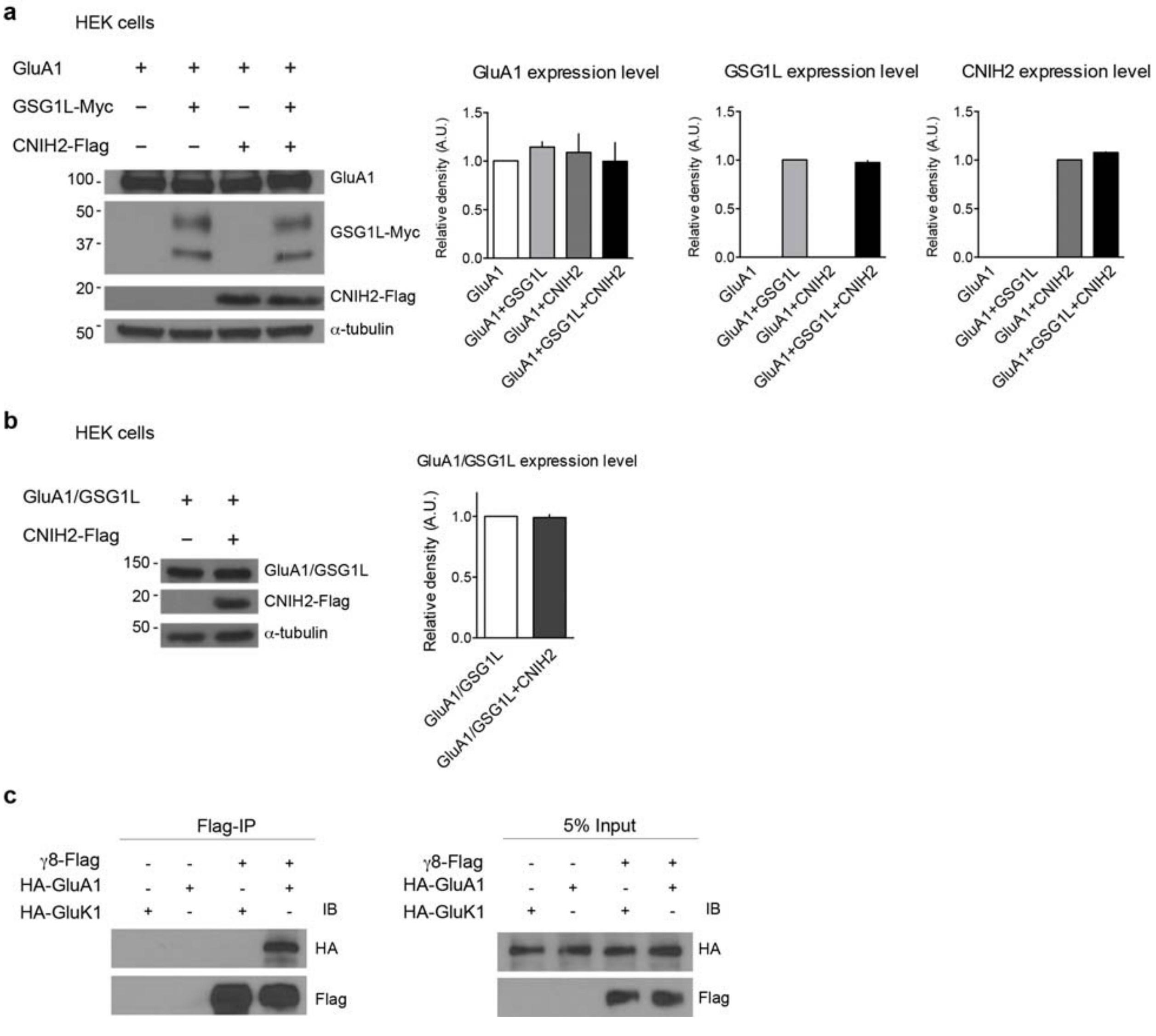

Supplementary Fig. 8. Characterization of protein expression of plasmids used in AMPAR kinetics assay.

(a) Western blot analysis of GluA1, GSG1L and CNIH2 expression level in HEK cells. There was no difference in GluA1 expression when co-expression GluA1 with CNIH2, GSG1L or both CNIH2 and GSG1L (n = 3, One-Way ANOVA analysis, *p* = 0.84). No difference in GSG1L (n = 3, *t*-test, *p* = 0.50) or CNIH2 expression (n = 3, *t*-test, *p* = 0.25) was detected.

(b) Western blot analysis of GluA1/GSG1L expression level in HEK cells. Co-expression with CNIH2 did not affect GluA1/GSG1L expression (n = 3, *t*-test, *p* = 0.99).

(c) HA-GluA1 or HA-GluK1 and γ8-Flag were expressed alone or together in HEK cells. Flag-tagged γ8 in cell lysates was immunoprecipitated with an anti-Flag antibody and immunoprecipitates (IP) were subjected to a western blotting assay with anti-HA and anti-Flag antibodies, respectively (left). 5% of the lysate used for immunoprecipitation (input) was also probed with indicated antibodies to confirm protein expression (right).

All data were presented as mean ± SEM.

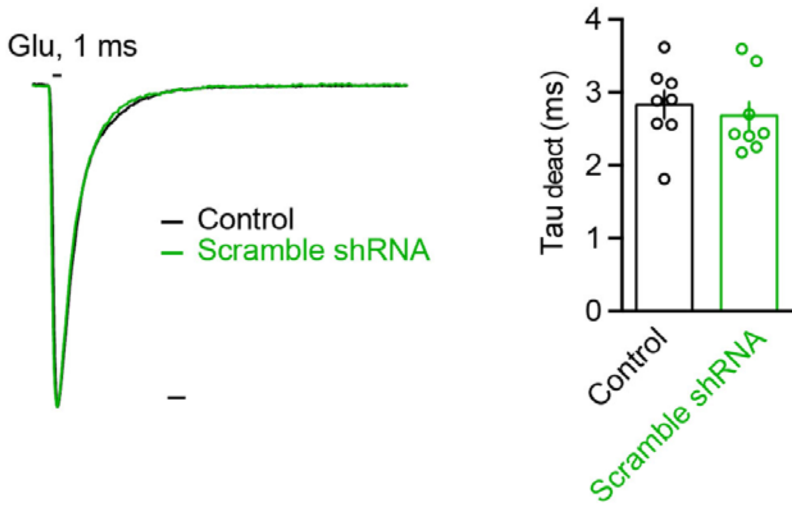

Supplementary Fig. 9. Expression of a scramble shRNA in CA1 neurons in mouse hippocampal slice cultures did not change AMPAR deactivation kinetics (weighted deactivation time constant: n = 8 for each; $p = 0.57$; *t*-test). Peak normalized sample traces are shown (left). All data were presented as mean ± SEM**.** Scale Bar: 2 ms.

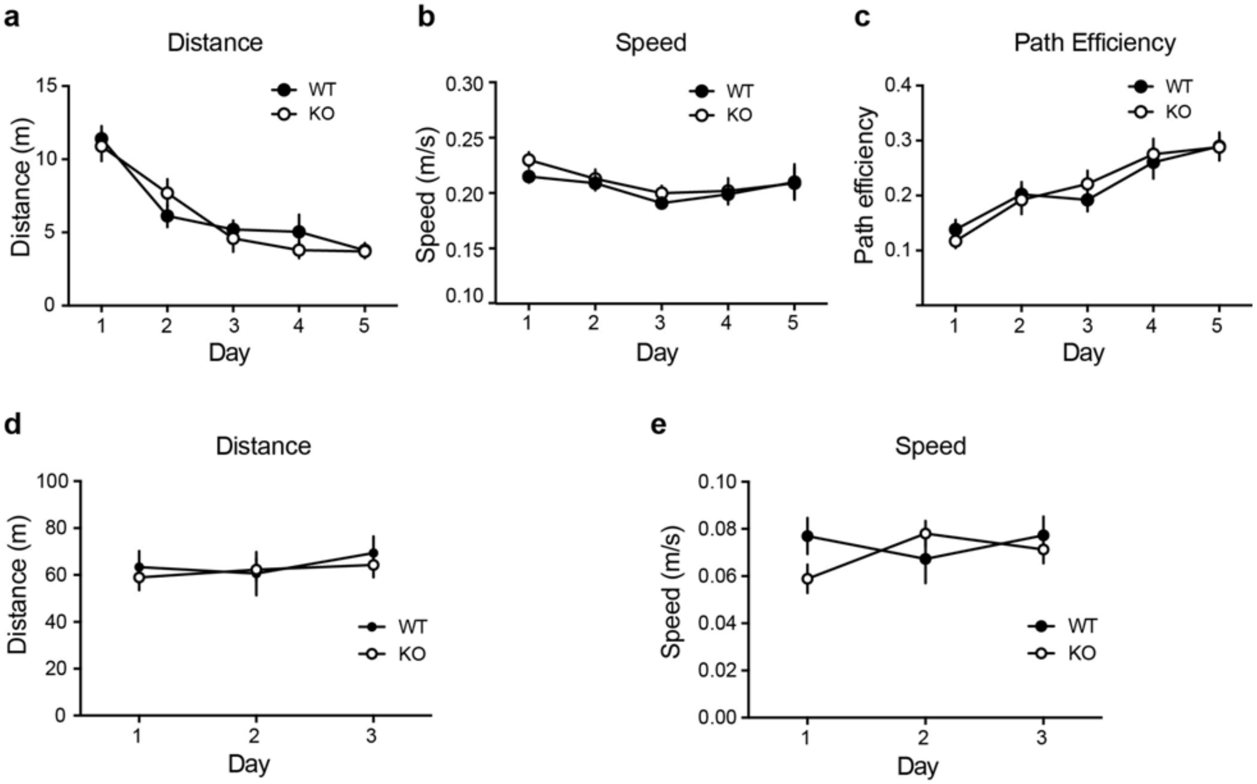

Supplementary Fig. 10. Behavioral characterization of GSG1L KOs.

(a-c) Morris water maze acquisition for WT (+/+, n = 18) and GSG1L KO (-/-, n = 19) mice consisted of four trials per day. Distance, speed and path efficiency were calculated by averaging data across trials within the same day. There was no difference in average distance travelled (a, $p = 0.74$), swim speed (b, $p = 0.33$) or path efficiency (c, $p = 0.88$) during trials in each day.

(d-e) Average distance and speed of the animal in the open-field box were calculated during habituation days in the object recognition memory test (+/+, n = 9; -/-, n = 9). There was no difference in average distance travelled (d, $p = 0.63$) and moving speed (e, $p = 0.46$) during each day.

All analyses were performed as 2-way ANOVAs with the between-subjects factor of Strain and the within-subjects factor of Day, and data were presented as mean ± SEM.

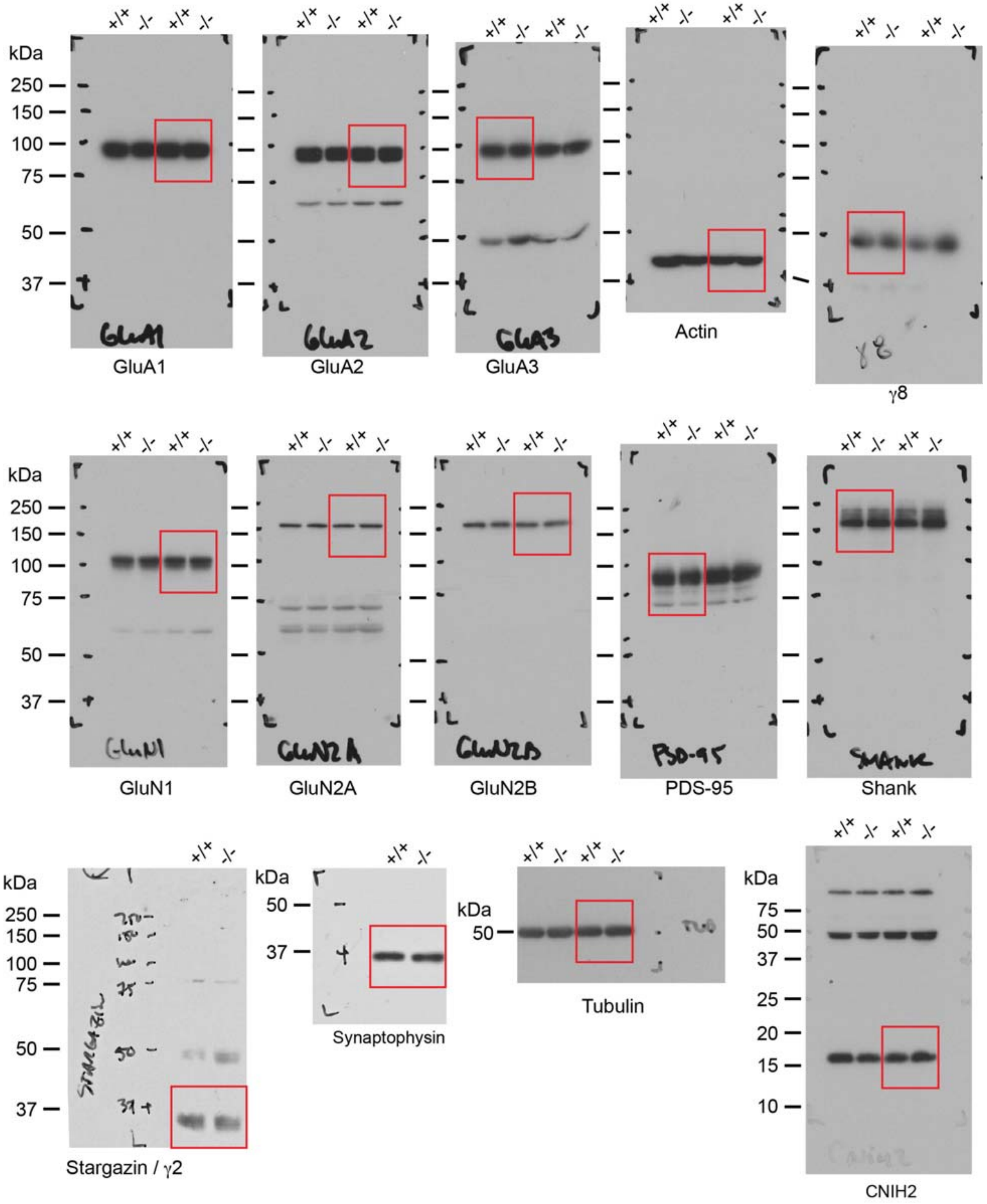

Supplementary Fig. 11. Uncropped scans of western blots shown in Figure 4d. Boxed areas in red indicate the cropped regions.

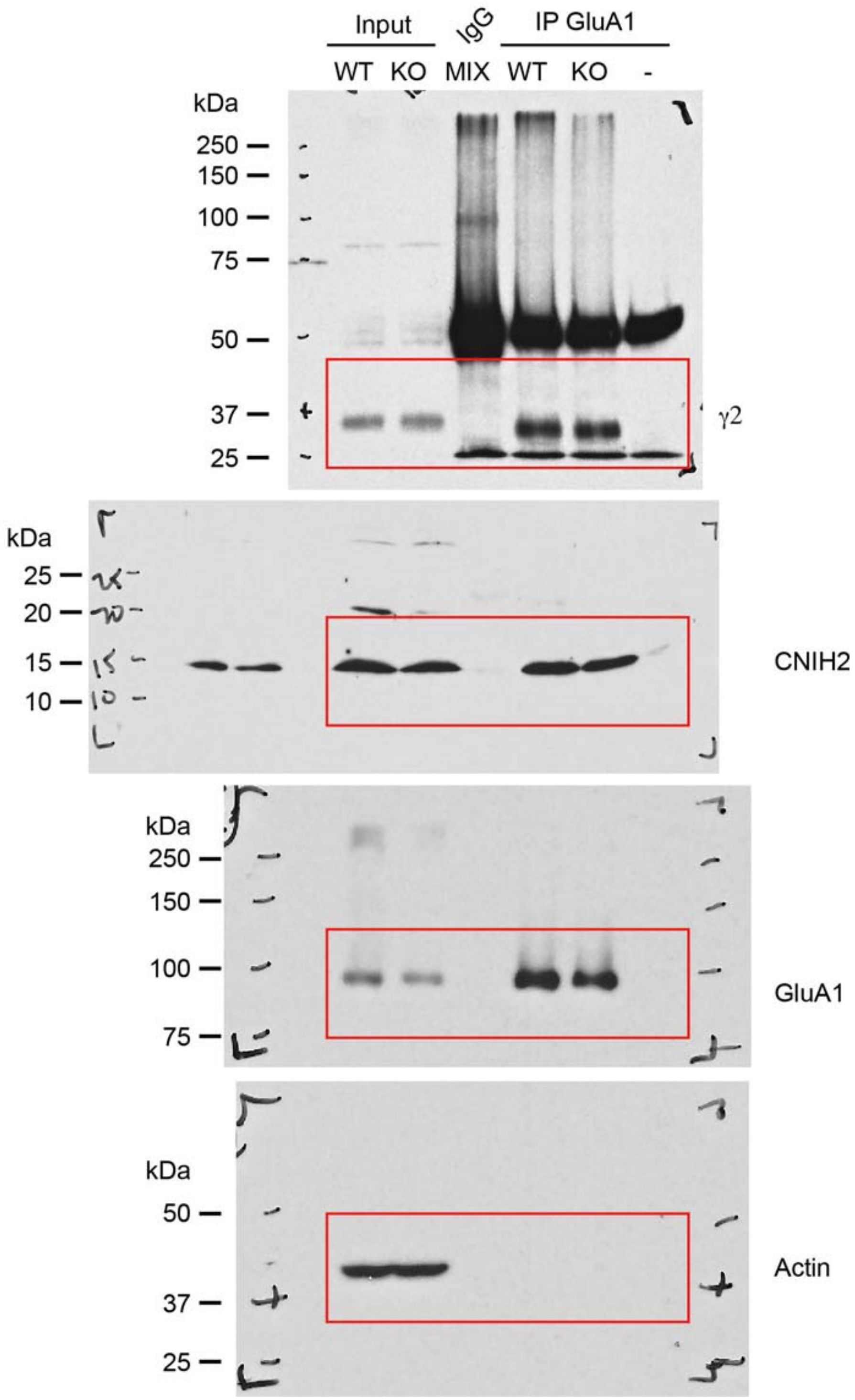

Supplementary Fig. 12. Uncropped scans of western blots shown in Figure 4e. Boxed areas in red indicate the cropped regions.

#

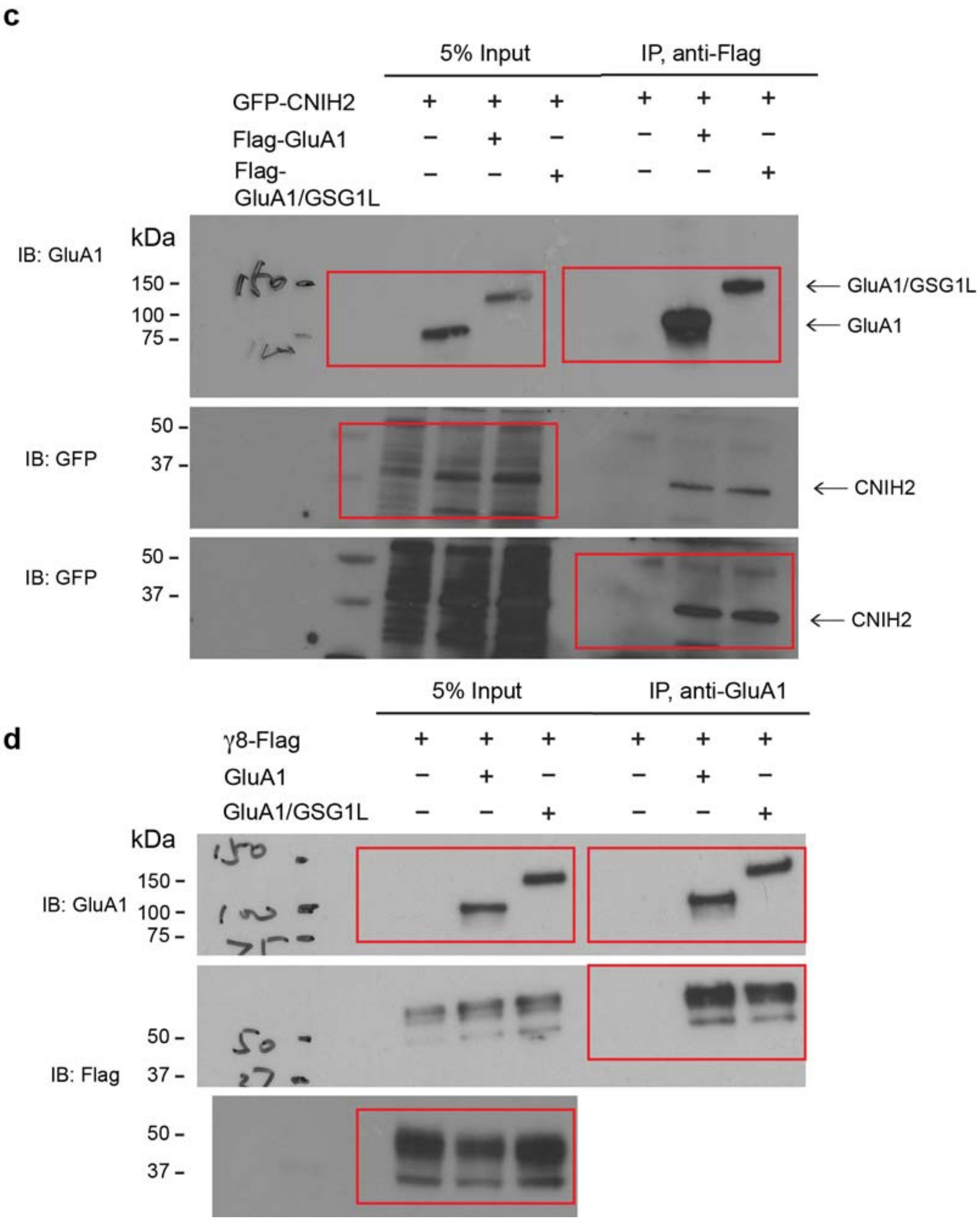

Supplementary Fig. 13. Uncropped scans of western blots shown in Figure 7c-d. Boxed areas in red indicate the cropped regions.

a

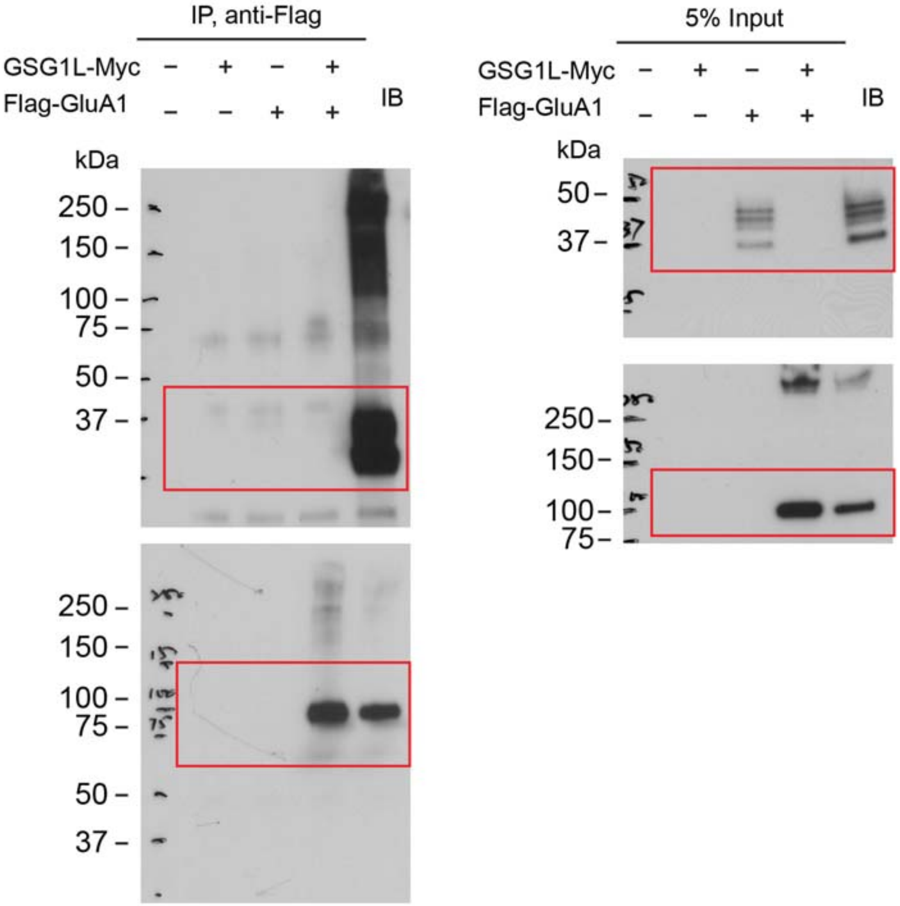

b

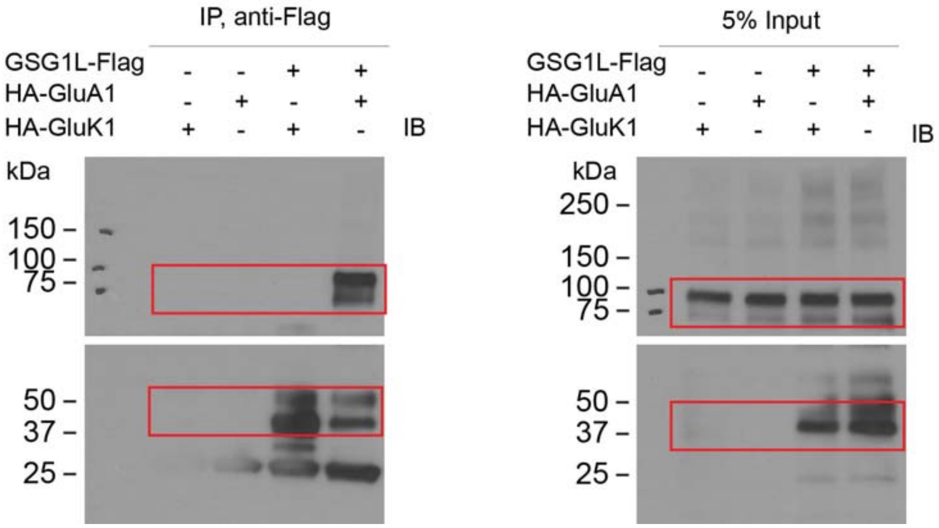

Supplementary Fig. 14. Uncropped scans of western blots shown in Supplemental Fig. 1a-b. Boxed areas in red indicate the cropped regions.

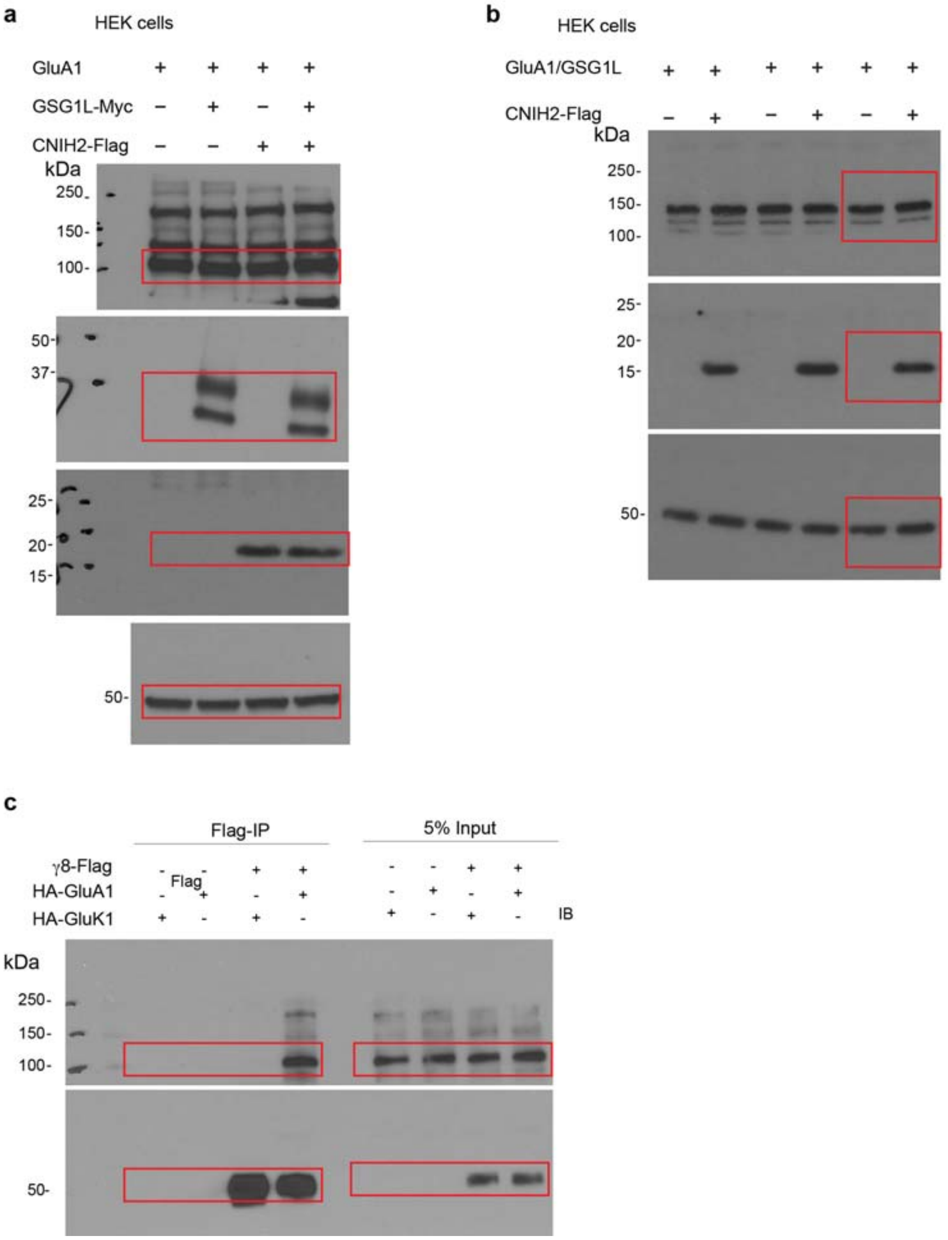

Supplementary Fig.15. Uncropped scans of western blots shown in Supplemental Fig. 8a-c. Boxed areas in red indicate the cropped regions.
